# Analysis of Gene Regulatory Networks of Taro (*Colocasia esculenta* (L.) Schott.) Soluble Starch Synthase Based on DeGN and KASP Marker Development

**DOI:** 10.1155/ijog/9953367

**Published:** 2025-03-01

**Authors:** Lu Jiang, Jianmei Yin, Li Wang, Xiaoyong Han, Peitong Zhang

**Affiliations:** Institute of Industrial Crops, Jiangsu Academy of Agricultural Sciences, Nanjing, China

**Keywords:** regulatory network, SNP, starch synthesis, taro, transcription factor

## Abstract

Taro (*Colocasia esculenta* (L.) Schott.) is an important edible and economically valuable crop that is also a source of high-quality starch. Its quality is determined by the content and proportion of amylopectin. Based on transcriptome sequencing of corms at different growth stages (T1–T6), 34,603 transcripts and 1727 novel genes with functional annotation were obtained. In total, 11,865 differentially expressed genes (DEGs) were identified among six development stages, with 3836 and 3404 DEGs in T2 versus T3 and T3 versus T4, respectively. The regulatory network of taro starch synthesis was constructed on the DeGNServer. Among three cloned soluble starch synthase (SS) genes, *CeSS II* might be the key gene responsible for soluble starch synthesis in taro corm. The putative transcription factor *CeMyb108* might play a negative role in starch synthesis. Sanger sequencing *CeSS II* gene revealed a single nucleotide polymorphism (SNP) between two variety groups with high and low starch content. A kompetitive allele-specific PCR (KASP) marker, namely, CeSS II-SNP, was developed and validated in a natural population of 89 taro accessions. The starch content of the C:T group amounts to 517.45 mg/g, which is significantly (22.3%) higher than its counterpart (T:T). This newly developed marker is proved to be effective and would facilitate marker-assisted breeding for taro with high starch content.

## 1. Introduction

Taro (*Colocasia esculenta* (L.) Schott) is a perennial monocotyledon belonging to the Araceae family; it has underground corms and is cultivated annually. It mainly originated in China, India, and Malaysia, among other regions. According to the Food and Agriculture Organization of the United Nations [1], total taro production ranks fifth among tuberous crops, following potato, cassava, sweet potato, and yam. The total taro production in China ranks second in the world. The main edible part of taro is an underground corm, which is rich in starch and accounts for 60%~70% of the total dry matter. It is often considered as a staple food in Asia, Africa, and India [2]. Its unique and appealing taste is directly related to the taro starch quality [3]. Compared with potatoes and sweet potatoes, taro starch has smaller starch particles, higher amylopectin content, and better cold and heat tolerance. Due to unique physical and chemical properties, taro starch has been widely used in baby food [4] and food thickener or adhesive [5–7] in the food industry.

Starch synthesis is a complex process that is catalyzed by a series of enzymes [8–10]. An earlier study on taro leaves used the transcriptome sequencing (mRNA) approach to identify the putative genes involved in starch biosynthesis in taro and reported 26 genes, such as starch branching enzyme A, soluble starch synthase I (SSI) and II (SSII), and UDP-glucose dehydrogenase [11]. Another study reported the identification and cloning of an ADP-glucose pyrophosphorylase and confirmed that its higher expression is positively correlated with higher starch contents in taro corms [12]. Among them, soluble starch synthase (SS) is an important component of starch synthase and is the core enzyme of plant biosynthesis of starch [13]. Current research on the mechanism of starch synthesis mainly focuses on *Arabidopsis*, rice, wheat, and other plant species [14, 15]. SS plays a vital role in the formation of starch branching, starch granules, and the degree of amylose polymerization [16] and has a very significant positive correlation with the accumulation rate of total starch and amylopectin [17]. Additionally, it plays a decisive role in the formation of rice quality [18]. SS mainly includes four subtypes: SS I, SS II, SS III, and SS IV, where SS II can be divided into SSS IIa and SSS IIb [19]. Our group cloned three different types of taro *SS* gene, named *CeSS I*, *CeSS II*, and *CeSS III*. It was found that these three genes were expressed significantly higher in corm than in other tissues (leaf, petiole, and root) and among which *CeSS II* was the highest. There was an overall positive correlation between *CeSS* expression and starch content. At present, it indicates that the *CeSS* plays a key role in taro starch synthesis [20]. Different soluble starch synthase subtypes play critical roles in different tissues, and it is more likely that they coordinate with each other and regulate starch synthesis.

In recent years, the rapid development of high-throughput sequencing technology, genomics, and systems biology has advanced network analysis algorithm and its application in studying complex biological processes and functions [21]. Through genome-based gene network (DeGN) analysis, the key regulatory factor *ZmNLP15* of nitrogen utilization at the seedling stage was found, which expanded the direction of regulation research for the theoretical gene of maize nitrogen efficient utilization [22]. Moreover, NAC36 transcription factor was coexpressed with starch synthesis–related genes in the maize endosperm [23]. *ZmEREB94* regulated SS *I* gene and functioned as an important regulatory factor in the starch synthesis pathway in maize [24]. DeGN based on gene interaction is an effective approach to analyze the taro *CeSS* gene regulatory network. The genome sequences provide valuable platform to identify numerous single nucleotide polymorphism (SNP) variations. They can be converted into kompetitive allele-specific PCR (KASP) markers and offer a high-throughput option for detection of agronomic traits of wheat, maize, and so on [25, 26]. The assembled taro chromosomal-level genome (XH01, 2n = 28), laid solid database for marker development [27].

Taro hybrid breeding has developed rapidly in many countries such as India, Bangladesh, and Japan [28–31]. Molecular markers such as amplified fragment length polymorphism (AFLP) [32], simple sequence repeat (SSR) [33, 34] and SNP [35] have been developed for taro. Quero-García et al. [36] reported the first genetic maps and QTL for yield traits in taro (*Colocasia esculenta* (L.) Schott). Two genetic linkage maps of taro were constructed using SNP and microsatellite markers [37]. QTLs for resistance to taro leaf blight and *Phytophthora* were identified [38, 39]. In this study, we constructed the regulatory network for the key enzyme genes (*CeSS*) in taro starch biosynthesis pathway, excavated the transcription factors that play key regulatory roles, analyzed its regulatory mode of starch synthesis in taro growth cycle, mined SNP loci, and developed KASP marker for high starch content breeding. The results would provide theoretical support for a better understanding of the molecular mechanism underlying taro starch synthesis and contribute a breeding marker for high starch content selection.

## 2. Materials and Methods

### 2.1. Plant Materials

XH01, as the reference genome been assembled earlier, is a well-known local taro variety in Jiangsu Province. It was planted in the Liuhe Animal Science Base of the Jiangsu Academy of Agricultural Sciences on March 31 and was harvested on November 2, 2020. XH01 plants sprouted on May 6 (30 days after sowing). Totally, 18 corm samples were collected at six growth stages with three replicates each: on the 30th day (June 5, T1), the 60th day (July 5, T2), the 90th day (August 4, T3), the 120th day (September 3, T4), the 150th day (October 3, T5), and the 180th day (November 2, T6) after the sprout. Ten grams of these corm samples was rapidly frozen with liquid nitrogen and stored at −70°C, which were prepared for transcriptome analysis. In total, a collection of 89 taro accessions were planted in the same field trial together with XH01. These taro varieties were collected from different provinces in China, including local varieties, breeding varieties, and wild resources (Table S1). Two-gram fresh leaves of 89 taro accessions were harvested and stored at −70°C. The corms were harvested for starch quality test and used to verify the newly developed KASP markers.

### 2.2. Transcriptome Sequencing and Gene Prediction and Annotation

Eighteen mRNA libraries of XH01 corm were constructed and sequenced. Total RNA was extracted by Plant RNA Kit R6827-01 (OMEGA Bio-tek) from the above 18 samples. One-microgram total RNA per sample was used as input material for the RNA sample preparations. Sequencing libraries were generated using NEBNext Ultra RNA Library Prep Kit for Illumina (NEB, United States) following manufacturer's recommendations, and index codes were added to attribute sequences to each sample. Briefly, mRNA was purified from total RNA using poly-T oligo-attached magnetic beads. Fragmentation was carried out using divalent cations under elevated temperature in NEBNext First-Strand Synthesis Reaction Buffer (5X). First-strand cDNA was synthesized using random hexamer primer and M-MuLV Reverse Transcriptase. Second-strand cDNA synthesis was subsequently performed using DNA Polymerase I and RNase H. The library fragments were purified with AMPure XP system (Beckman Coulter, Beverly, United States). The clustering of the index-coded samples was performed on a cBot Cluster Generation System using TruSeq PE Cluster Kit v4-cBot-HS (Illumina) according to the manufacturer's instructions. After cluster generation, the library was sequenced on an Illumina platform and paired-end reads were generated. Clean data with high quality was obtained by filtering raw data, which removes adapter sequence and reads with low quality. These clean data were further mapped to predefined reference genome with HISAT2 [40]. StringTie [41] was used to compare and splice the results, and ASprofile [42] was used to obtain the variable splicing type and corresponding expression amount of each sample. At the same time, the original genome annotation information was compared to discover new transcripts and taro genes. DIAMOND [43] was performed to annotate the predicted genes against nr (NCBI), TrEMBL [44], Swiss-Pro (http://www.uniprot.org/), COG (http://www.ncbi.nlm.nih.gov/COG/), KOG (http://www.ncbi.nlm.nih.gov/KOG/), KEGG (http://www.genome.jp/kegg/), and eggNOG (http://eggnog-mapper.embl.de). InterProScan [45] and HMMER [46] software were used to annotate the functions of GO (http://www.geneontology.org/) and Pfam (http://pfam.xfam.org/), respectively.

### 2.3. Screening for DEGs

The gene expression level estimation method [47] was used to estimate the number of fragments (FPKM, fragments per kilobase of transcript per million fragments mapped) of transcriptional sequence per thousand base pairs sequenced per million base pairs for readcount. Principal component analysis (PCA) was performed to evaluate the indices of biological repetition correlation using BMKCloud (http://www.biocloud.net/). The differentially expressed genes (DEGs) were screened using DESeq2 [48] with false discovery rate (FDR) < 0.01 and fold change ≥ 2 as the threshold. The BioProject ID of the transcriptome sequence data in the China National GeneBank DataBase is CNP0005208.

### 2.4. Regulatory Network Construction

The DeGNServer (https://www.zhaolab.org/DeGNServer/) was used to construct a taro gene network through gene interactions [49]. Expression data of 18 taro corm DEGs from T1 to T6 was converted to TAB-delimited text format and submitted to DeGNServer. Value-based coexpression method was used to construct gene network. Similarities were measured by Pearson correlation coefficient. Regulator interactions were identified by computing pairwise mutual information (MI value) among all genes. The *CeSS* gene was used as a node to capture the interacting genes and build the subregulatory network. According to the magnitude of gene interactions and gene function annotation, the transcription factors involved in regulation were screened from the subregulation network. We then analyzed the structure and target genes of candidate transcription factors (http://planttfdb.cbi.pku.edu.cn/, http://www.sbg.bio.ic.ac.uk/phyre2/html/page.cgi?id=index) and screened candidate transcription factors that regulate starch synthesis.

### 2.5. RT-qPCR Analysis

To validate candidate genes selected from the network analysis, expression levels were determined by quantitative RT-qPCR. The reaction procedure was as follows: 95°C for 30 min, 45 cycles of 95°C for 5 s, 58°C for 30 s, and 72°C for 30 s. Through the dissolution curve of the quantitative reaction, the specificity of the primer was verified to meet the test requirements. RT-qPCR reaction was performed in the LightCycler 480 fluorescent RT-qPCR detection system (Roche, Switzerland), and the relative expression of genes was calculated according to the 2^-*ΔΔ*CT^ algorithm [50]. The primers used for RT-qPCR were designed using Primer 5.0 software, with *CeSS I* (EVM0025803), *CeSS II* (EVM0005345), *CeMyb108* (EVM0003916), and *Ser/thr kinase* (EVM0022436) (Table 1).

### 2.6. Starch Content Determination

The dried corms of 89 taro resources were selected for starch quality test. The main indexes of starch quality include total starch content, amylose content, amylopectin content, and amylopectin/amylose content ratio. The amylose content and amylopectin content in taro were quantified with the dual-wavelength method following the previous reports [51–53]. Measurements were repeated in triplicate. Data was collected, collated, and analyzed using Excel 2016 software and SPSS 11.0 software.

### 2.7. SNP Mining and Marker Development

DNA of 89 natural taro genotypes was isolated from leaves using the cetyltrimethylammonium bromide (CTAB) method [54]. Five taro varieties including both high and low starch content were selected for Sanger sequencing of *CeSS II*. Multiple sequence alignment was conducted by MAFFT v7.427 (http://mafft.cbrc.jp/alignment/software/). SNPs were detected and developed to KASP markers for genotyping [55]. Primer sequences were F1: GAAGGTGACCAAGTTCATGCTGACCTTAGCTGGGATCATGCC, F2: GAAGGTCGGAGTCAACGGATTGGACCTTAGCTGGGATCATGCT, and R: AACAAGGACTTCCTCATATAGCTGAGC.

#### 2.7.1. KASP Reaction

Each reaction mixture (10 *μ*L) consisting of 1.25 *μ*L DNA (50 ng/L), 2.5 *μ*L 2 KASP master mix, and 1.25 *μ*L primer mix (0.5 *μ*L of each allele-specific primer and 0.75 *μ*L of the common primer) was amplified in the CFX Connect Real-Time System (Bio-Rad, United States). The reaction mixtures were initially denatured at 95°C for 10 min prior to 37 cycles of 95°C for 20 s and 55°C for 1 min. Fluorescence signals were read at 25°C for 30 s. The Python's scipy package for the KW test (i.e., Kruskal–Wallis test) (https://scipy.org/) was used to analyze the correlation between traits (starch content) and KASP marker in 89 taro genotypes.

## 3. Results

### 3.1. Transcriptome Analysis and Gene Function Annotation

Through transcriptome sequencing of 18 samples, 126.29 GB of data were obtained. Clean data of each sample reached 5.99 Gb, and the Q30 base percentage was 91.46% and above. Compared with the reference genome, the mapping rate of T1–T6 samples was 74.25%~88.68%, and 34,603 transcripts were obtained (Table S2). A total of 5908 new genes were discovered, and 1727 new genes with functional annotation were obtained after variable splicing prediction analysis, gene structure optimization, and new gene discovery (Table 2).

### 3.2. Identification of DEGs

**Figure 1:**
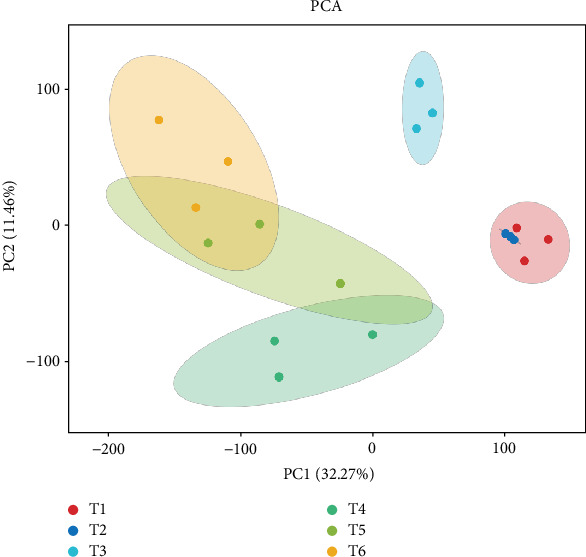
Correlations among the samples of taro corm development stages from T1 to T6. PCA: principal component analysis. The *x*-axis and *y*-axis showed that the first two principal components (PC1 and PC2) separated 18 samples, accounting for 32.27% and 11.46% of the total variability, respectively. This figure was performed using BMKCloud (http://www.biocloud.net/).

**Figure 2:**
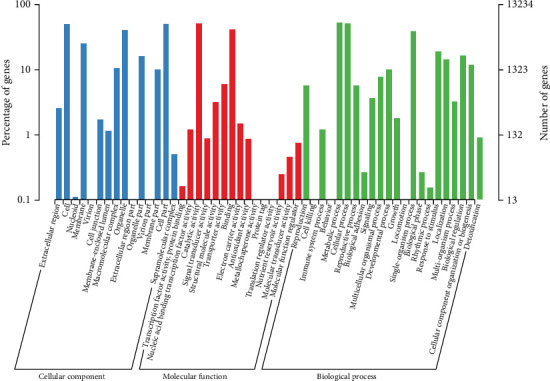
GO functional annotation of DEGs. The *x*-axis was function class; the left *y*-axis was the percentage of gene numbers and the right was gene numbers. This image shows the genetic enrichment of the secondary functions of GO.

PCA showed a good correlation among replicates (Figure 1). T1 and T2 samples were relatively closer, which indicates similar profile of gene expression at the taro corm early development. A total of 13.234 DEGs were found. The DEGs were annotated and enriched on GO database (Figure 2). Among them, the differential genes are mainly concentrated in the biological processes of metabolism and cells. The T2 to T4 period is an important period for the development and expansion of taro corms and the accumulation and enrichment of substances and is also a period of rapid change in expression profiles of responsible genetic factors. By comparing gene expression profiles between different growth stages, we found that there are 3836 and 3404 DEGs in T2 versus T3 and T3 versus T4, respectively.

### 3.3. Construction of Taro Starch Synthesis Regulation Network

Analysis of genome-scale gene networks using large-scale gene expression data provides unprecedented opportunities to uncover gene interactions and regulatory networks involved in various biological processes and developmental programs, leading to accelerated discovery of novel knowledge of various biological processes, pathways, and systems. Based on the transcriptome data of DEGs from T1 to T6, a value-based coexpression network was constructed via the DeGN model, with Spearman's rank correlation estimation algorithm, which was used to set the interaction threshold of genes to 0.9, forming the interaction network between genes (Table S3). There were three subtypes of taro soluble starch synthase (*CeSS*): *CeSS I*, *CeSS II*, and *CeSS III*. *CeSS I* and *CeSS II* were DEGs during the development of taro corm. With these two genes as seeds, a subregulatory network consisting of 159 genes and 2146 interactions was constructed (*p* > 0.95).

**Figure 3:**
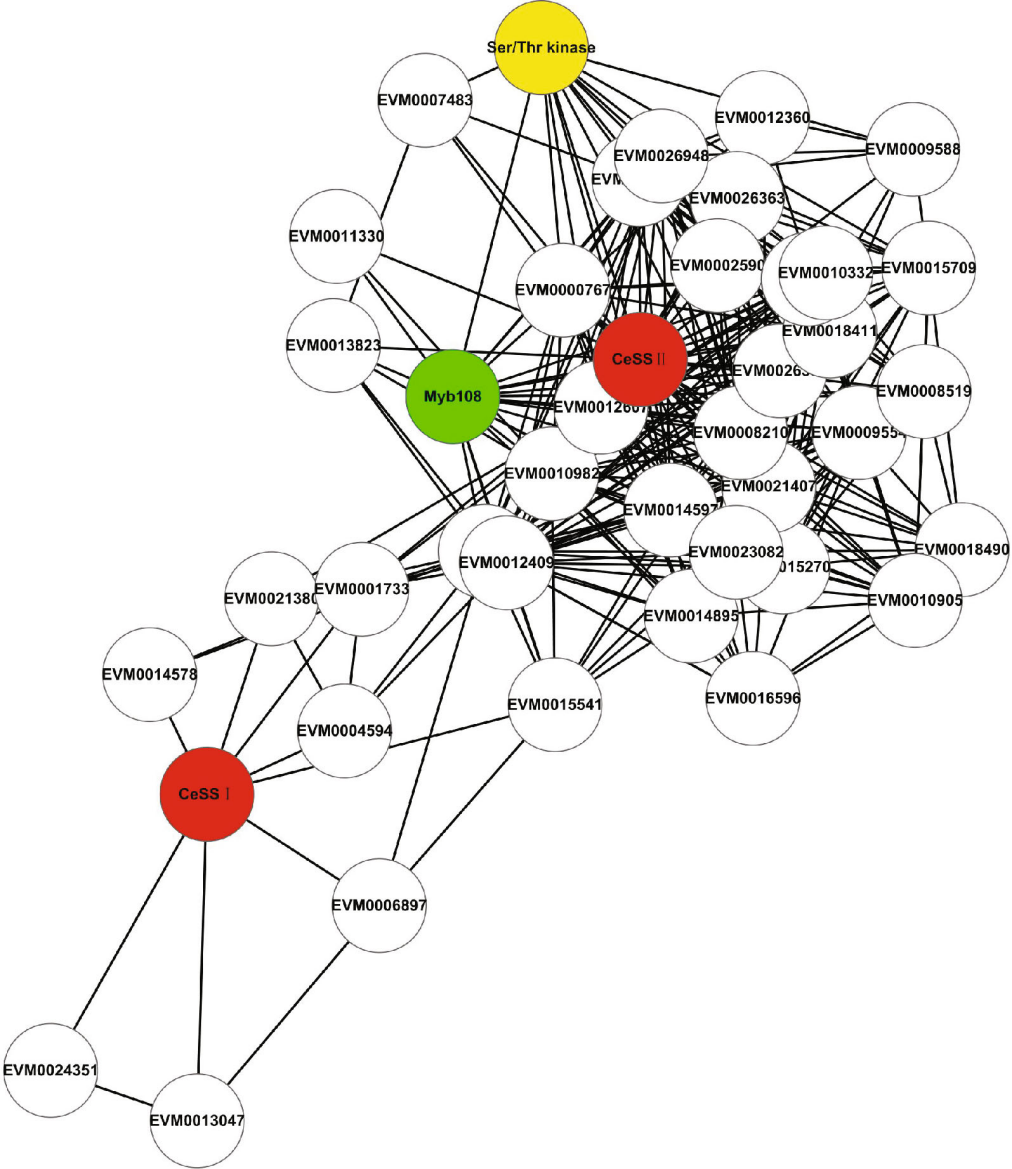
Schematic diagram of gene interactions. Red nodes: core genes CeSS II (EVM0005345) and CeSS I (EVM0025803). Green node: transcription factor CeMyb108 (EVM0003916). Yellow node: kinase-related gene Ser/thr kinase (EVM0022436).

The coexpression network was analyzed and displayed by Cytoscape v2.8 [56], and *CeSS* genes as the cores were used to capture adjacent genes screened, and the interaction diagram of the first-neighbor genes was generated (Figure 3 and Table S6). It was found that the transcription factor *Myb108* (EVM00003916) was related to the core genes *CeSS II* (EVM0005345) and *CeSS I* (EVM0025803) and the kinase-related gene *Ser/thr kinase* (EVM0022436).

**Figure 4:**
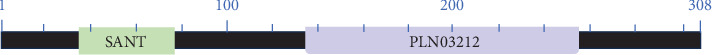
Schematic diagram of CeMy108. SANT: transcription factor binding site; PLN03212: transcriptional inhibition structure.

The transcription factor *CeMyb108* was mined by NCBI CDART to obtain a conserved transcriptional negative regulatory domain (Figure 4). The 2–4 kb upstream sequences of *CeSS I* and *CeSS II* genes, presumed promoters, were obtained from the taro genome XH01, and two DNA binding sites of Myb, ⁣′GTTGGCCACCACCACTACTAT⁣′ and ⁣′CGTCGTCACCACCTTGTCG⁣′, were found by predicting promoter regulation through the PlantRegMap of the PlanTFDB database.

### 3.4. Expression Pattern of CeSS at Different Developmental Stages

**Figure 5:**
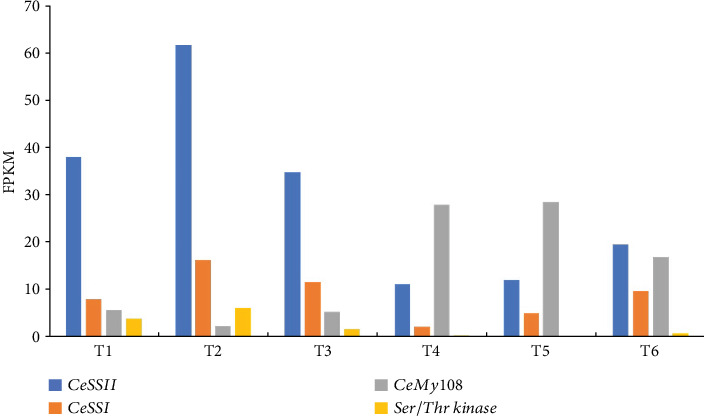
Expression pattern of soluble starch synthase and its related genes in taro. The *x*-axis represents six growth stages (T1–T6) and the *y*-axis represents gene expression level (FPKM, fragments per kilobase of transcript per million fragments mapped).

According to the transcriptome data during the developmental stages of taro corm, *CeSS I*, *CeSS II*, and *Ser/thr kinase* have a similar expression pattern (Figure 5). The expression level of *CeSS I*, *CeSS II*, and *Ser/thr kinase* was highest at the T2 stage and then exhibited a downward trend. At the T4 stage, it decreased to its lowest level, and at the T5 to T6 stage, it rebounded slightly. The abundance of *CeSS II* was significantly higher than that of *CeSS I* so that *CeSS II* was a subtype with a higher expression level of soluble SS in taro corm. The transcription factor *CeMyb108*, which was found by coexpression analysis, showed an opposite expression pattern.

Through quantitative analysis and verification of the expression abundance and pattern of four genes (Table S4), CeSS II may be the main gene controlling the soluble starch synthesis in taro corm. The expression rapidly increased in the early stages of taro corm formation and reached its maximum value at the rapid expansion stage and gradually decreased at the matured stage. Therefore, CeMyb108 might affect the soluble starch synthesis of taro as taro corm develops.

### 3.5. The Starch Content of Taro Germplasm Resources

According to the analysis of the starch quality of 89 taro germplasm resources, the amylose content (on a dry basis) ranged from 25.29 to 211.46 mg/g, the amylopectin content (on a dry basis) ranged from 135.28 to 646.95 mg/g, and the ratio of amylopectin/amylose content ranged from 0.86 to 9.70. The starch contents varied greatly among different taro genotypes. Three samples with high starch content (V05, V21, and V24) and two samples with lower starch content (V72 and V816) were selected for marker development (Table S1).

### 3.6. Marker Development and Polymorphic Identification

**Figure 6:**
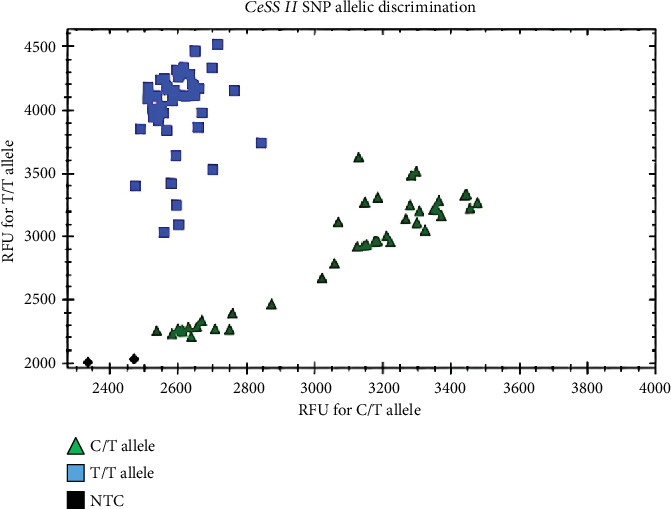
Genotypes of 89 taro resources by kompetitive allele-specific PCR (KASP) assay. The *x*-axis was relative fluorescence units (RFU) for C:T allele; the *y*-axis was RFU for T:T allele. The blue squares and green triangles represent the homozygous (T:T) and heterozygous (C:T), respectively. The black squares represent no template control, NTC.

Based on sequencing results, a SNP C:T on 567th of CeSS II was detected between the high and low starch varieties. The KASP categorized 89 taro accession into two major groups, 45 C:T type and 44 T:T type (Figure 6, Table S5). Based on the KW test, two groups are significantly different from each other with regard to four phenotypes: amylose content, amylopectin content, starch content, and the ratio of amylopectin/amylose content (*p* < 0.05) (Table 3).

## 4. Discussion

### 4.1. Starch Content in Taro Corm

The proportion of plant starch varies greatly. The content of amylose is 20%–25% and that of amylopectin is 45%–55% in common grains such as wheat and corn. The amylose content of rice is slightly lower than that of wheat, while the amylopectin content of glutinous rice and glutinous corn can reach 99% [57]. The amylopectin content of taro is higher in common plants, and the starch components and the content and proportion of amylopectin vary greatly depending on the variety [11]. Eighty-nine resources are collected from different provinces in China. They showed significant differences in plant phenotype, taro morphotypes, and starch contents. The amylose content (on a dry basis) ranged from 25.29 to 211.46 mg/g, the amylopectin content (on a dry basis) ranged from 135.28 to 646.95 mg/g, and the ratio of amylopectin/amylose content ranged from 0.86 to 9.70. Taste and flavor are important indicators for evaluating high-quality taro, while novel varieties with high amylopectin content are the goal of taro modern breeding. Mining the regulator of starch synthesis can facilitate better understanding the process of starch synthesis and usage of taro.

### 4.2. Expression Profiles of CeSS Gene

The process of taro corm maturity is the process of starch synthesis, transportation, transformation, and accumulation in the corm, which plays a vital role in the taro cycle and biosynthesis. Among them, amylopectin, which accounts for a higher proportion of starch, determines the quality of taro starch. However, at present, taro starch research has focused on content determination, quality analysis, and the cloning and separation of a small amount of synthase. Little is known about its expression, synthesis, and regulation mode. Through transcriptome analysis of taro leaves, 26 genes related to starch synthesis were identified [11]. Whole-transcriptome sequencing was used to screen for differentially expressed RNA, including mRNAs, CircRNAs, and miRNAs, and a total of 11,203 mRNAs, 245 CircRNAs, and 299 miRNAs were obtained in taro [58]. SSI and SSII genes were isolated from taro (*Colocasia esculenta* var. *esculenta*) tubers [59, 60]. More SSI transcripts were detected in taro leaves than in tubers, while no transcript was observed in petioles. A large amount of SSII was detected in senescence leaves and tubers. DeGN is an effective method for analyzing the spatial and temporal expression patterns of the regulatory network of taro starch synthesis. In this study, through quantitative analysis of expression of the CeSS family members, we found that *CeSS II* was highly expressed in corm development. Many genes were expressed in the early stage of corm development, where they played key roles. The expression of genes gradually decreased as the corm matured, which synchronize with the expansion of taro corm.

### 4.3. Potential Role of Myb in Starch Synthesis

The Myb family is one of the largest and most functional transcription factor families in eukaryotes. It plays an important role in the plant cell cycle, cell morphogenesis, growth and development, hormone response, biotic and abiotic stress resistance, and secondary metabolites [61]. In *Panax notoginseng*, PnMyB4 negatively modulates saponin biosynthesis through *PnMYB1* [62]. *ZmMyb14* was tested to raise the promoter activity of *ZmSh2*, *ZmBt2*, *ZmGBSSI*, *ZmSSI*, and *ZmSBE1* in maize endosperm via transient gene overexpression assays and also directly bind the promoters of starch synthesizing genes in yeast, which indicated it was a crucial regulator related to the biosynthesis of starch [63]. The role of Myb in starch synthesis regulation was widely studied in maize but was still unclear in rhizome crops. In taro, *CeMyb108* contains a conserved transcriptional negative regulatory domain and may play a negative role in the accumulation of starch synthesis. The mechanism of action may be different from maize, and further studies are needed.

### 4.4. KASP Assay

The KASP genotyping assay utilizes a unique form of KASP combined with a novel, homogeneous, fluorescence-based reporting system for the identification and measurement of genetic variation occurring at the nucleotide level to detect SNPs or inserts and deletions (InDels) [64]. KASP is the main SNP genotyping method and has the advantages of high accuracy, low cost, and high throughput [65, 66]. In this study, there were only two genotype categories of C:T and T:T. Taro, one of the world's oldest crops, has been domesticated for the edible quality. The index of quality selection is mainly based on taste, and taro taste quality is directly related to starch. Under long-term artificial selection, it may lead to a bias in the distribution of genotypes. There was a significant correlation between the KASP marker and starch traits. The result laid theoretical basis for molecular marker–assisted breeding in taro.

## 5. Conclusions

In this study, the excavation of taro starch synthesis key genes, the construction of an expression profile regulatory network, and the taro soluble SS gene *CeSS* and *Myb108* transcription factor were studied. The starch-related SNP sites were mined by resequencing to establish a set of KASP SNP markers for selecting high-quality starch cultivars. *CeSS II* might be the key gene controlling the soluble starch synthesis of taro in the corm, but *CeMyb108* may be playing a negative role in starch synthesis. CeSS II-SNP marker significantly contributed to increased starch content of 517.45 mg/g, which is 22.3% higher than its counterpart (T:T). By SNP genotyping of taro natural population or tissue culture mutation population, the single plant material with high starch content was quickly and accurately selected in the early stage of taro plant growth, so as to reduce the plant growth time and shorten the breeding cycle. Taro germplasm with high starch content was quickly selected from taro local varieties or resources, so as to carry out the effective protection and utilization of taro resources.

## Figures and Tables

**Table 1:** The information of RT-qPCR primers.

**Primers**	**Sequence (5**⁣′**-3**⁣′**)**	**Tm value (°C)**	**Product size (bp)**	**Annotation**
*CeSS I* (EVM0025803)	GAGTCAGAGGTGTTGCTCCC CGACCATGACAGATGACCA	58	301	K00703 starch synthase [EC:2.4.1.21] (A)

*CeSS II* (EVM0005345)	GGATCATGCCGCTCAGCTAT GGATCATGCCGCTCAGCTAT	58	247	K00703|0|pda:103707191|granule-bound starch synthase 2

*CeMyb108* (EVM0003916)	TGGCTTAGAGAACTGCAGCC TGGCTTAGAGAACTGCAGCC	60	264	K09422|5.41884e-116|pda:103712886|transcription factor MYB108-like

*Ser/thr kinase* (EVM0022436)	CTACACTGACGGCCAGAGG CTACACTGACGGCCAGAGG	58	251	Predicted: serine/threonine-protein kinase HT1

Actin	TCTGGCCACCACACCTTCTAC GACACACCGTCACCAGAGTC	60	226	K10355|pda:103720863 actin

*Note:* RT-qPCR primers were designed based on taro reference genome XH01 [27] and also were identified in XH01.

**Table 2:** Novel taro genes of function annotation.

**Annotated databases**	**New gene numbers**
COG	225
GO	1181
KEGG	943
KOG	704
Pfam	1007
Swiss-Prot	739
TrEMBL	1655
eggNOG	1287
nr	1537
All	1727

*Note:* Transcriptional profiles of 1727 new genes were found, and gene function was annotated based on the COG, GO, KEGG, KOG, Pfam, Swiss-Prot, TrEMBL, eggNOG, and nr databases.

**Table 3:** The KW statistic between the marker and phenotypes.

**Phenotypes**	**KW statistic**	**p** ** value**
Amylose content	17.17355	0.000034
Amylopectin content	12.68401	0.000369
Starch content	17.58406	0.000027
Amylopectin/amylose	7.762162	0.005335

*Note:* KW statistic: Kruskal–Wallis statistic. A *p* value less than 0.05 is considered as a significant association and 0.01 as highly significant.

## Data Availability

The authors confirm that the data supporting the findings of this study are available on the China National GeneBank DataBase (CNGBdb, https://db.cngb.org/) website with Accession No. CNP0005208.

## Supporting Information

Supporting Information Additional supporting information can be found online in the Supporting Information section. Table S1: List of 89 taro resources, original source, variety type, morphotype, phenotypic traits, and starch component contents. Table S2: All transcript expression value (FPKM) in taro corm developing stage T1 to T6. Table S3: Gene interactions between DEGs in taro corm developing stage. Table S4: Expression profile of *CeSSI*, *CeSS II*, *CeMy108*, and *SerThr kinase*. Table S5: The genotypes of 89 taro resources. Table S6: 159 node genes of *CeSS* regulatory network.
