# Supplementary material for: Analysis of Gene Regulatory Networks of Taro (Colocasia esculenta (L.) Schott.) Soluble Starch Synthase Based on DeGN and KASP Marker Development
Source: Int J Genomics. 2025 Mar 1;2025:9953367. doi: 10.1155/ijog/9953367 (PMC11991784; doi:10.1155/ijog/9953367)
Supplement: Supporting Information — Additional supporting information can be found online in the Supporting Information section. Table S1: List of 89 taro resources, original source, variety type, morphotype, phenotypic traits, and starch component contents. Table S2: All transcript expression value (FPKM) in taro corm developing stage T1 to T6. Table S3: Gene interactions between DEGs in taro corm developing stage. Table S4: Expression profile of CeSSI, CeSS II, CeMy108, and SerThr kinase. Table S5: The genotypes of 89 taro resources. Table S6: 159 node genes of CeSS regulatory network. [file 9953367.f1.zip › Supplementary Table S1.pdf]

Supplementary Table S1. List of 89 taro resources, original source, variety type, morphotype, phenotypic traits and starch component contents.

| ID | Name of taro varieties | Original source | Variety type | Morphotype | Shape of corm | Color of corm bud | Shape of cormel | Color of cormel bud | amylose content | amylopectin content | starch content | amylopectin / amylose |
|---|---|---|---|---|---|---|---|---|---|---|---|---|
| v01 | Baidayu | Taizhou Jingjiang, Jiangsu | native breed | multi-cormel | spherosome | yellowish white | ovoid | yellowish white | 74.47 | 427.74 | 502.21 | 5.74 |
| v02 | Danyang lvyayu | Zhenjiang Danyang, Jiangsu | native breed | multi-cormel | spherosome | yellowish white | ovoid | yellowish white | 57.91 | 393.30 | 451.21 | 6.79 |
| v03 | Haian xiangheyu 1 | Nantong Haian, Jiangsu | native breed | multi-cormel | spherosome | yellowish white | prolate ellipsoi | yellowish white | 117.07 | 520.02 | 637.09 | 4.44 |
| v04 | Haian xiangheyu 2 | Nantong Haian, Jiangsu | native breed | multi-cormel | spherosome | light red | prolate ellipsoi | light red | 109.01 | 528.07 | 637.09 | 4.84 |
| v05 | Hunan binlangyu | Hengyang Qidong, Hunan | native breed | single corm | spherosome | yellowish white | prolate ellipsoi | light red | 211.46 | 526.35 | 737.81 | 2.49 |
| v06 | Huanghezi | Taizhou Jingjiang, Jiangsu | native breed | multi-cormel | spherosome | yellowish white | ovoid | yellowish white | 37.27 | 361.41 | 398.69 | 9.70 |
| v09 | Jintan zigengyu | Changzhou Jintan, Jiangsu | native breed | multi-cormel | spherosome | yellowish white | ovoid | yellowish white | 109.99 | 437.26 | 547.25 | 3.98 |
| v10 | Jintan zilvgengyu | Changzhou Jintan, Jiangsu | native breed | multi-cormel | spherosome | yellowish white | prolate ellipsoi | yellowish white | 59.45 | 374.42 | 433.87 | 6.30 |
| v12 | Rudong bingfangyu 1 | Nantong Rudong, Jiangsu | native breed | multi-cormel | spherosome | light red | prolate ellipsoi | light red | 76.19 | 417.62 | 493.81 | 5.48 |
| v13 | Rudong bingfangyu 2 | Nantong Rudong, Jiangsu | native breed | multi-cormel | spherosome | light red | prolate ellipsoi | light red | 91.16 | 441.13 | 532.29 | 4.84 |
| v14 | Rudong haibianyu | Nantong Rudong, Jiangsu | native breed | multi-cormel | spherosome | yellowish white | prolate ellipsoi | yellowish white | 131.90 | 553.15 | 685.05 | 4.19 |
| v15 | Rugao xiangtangyu | Nantong Rugao, Jiangsu | native breed | multi-cormel | spherosome | red violet | turbinate | red violet | 125.18 | 560.10 | 685.28 | 4.47 |
| v17 | Suyu 1 | Taizhou Jingjiang, Jiangsu | bred varieties | multi-cormel | spherosome | red violet | prolate ellipsoi | red violet | 94.84 | 499.43 | 594.27 | 5.27 |
| v18 | Suyu 2 | Taizhou Jingjiang, Jiangsu | bred varieties | multi-cormel | spherosome | light red | ovoid | light red | 80.09 | 480.53 | 560.62 | 6.00 |
| v19 | Suyu 3 | Changzhou Jintan, Jiangsu | bred varieties | multi-cormel | spherosome | light red | prolate ellipsoi | light red | 131.33 | 552.08 | 683.41 | 4.20 |
| v20 | Suyu 4 | Taizhou Taixing, Jiangsu | bred varieties | multi-cormel | spherosome | light red | turbinate | light red | 109.65 | 525.67 | 635.32 | 4.79 |
| v21 | Suyu 5 | Nantong Haimen, Jiangsu | bred varieties | multi-cormel | ovoid | light red | prolate ellipsoi | light red | 163.50 | 646.95 | 810.45 | 3.96 |
| v22 | Suyu 6 | Nantong Rugao, Jiangsu | bred varieties | multi-cormel | spherosome | yellowish white | prolate ellipsoi | yellowish white | 57.64 | 396.46 | 454.10 | 6.88 |
| v23 | Sucaiyu 7 | Fuzhou Yongtai, Fujian | bred varieties | multi-cormel | spherosome | light red | ovoid | light red | 84.04 | 437.43 | 521.47 | 5.20 |
| v24 | Taiyu 1 | Taizhou Taixing, Jiangsu | bred varieties | multi-cormel | spherosome | light red | ovoid | light red | 129.86 | 578.39 | 708.25 | 4.45 |
| v27 | Taicang xinmaoyu 1 | Suzhou Taicang, Jiangsu | native breed | multi-cormel | spherosome | light red | prolate ellipsoi | light red | 62.35 | 375.43 | 437.78 | 6.02 |
| v28 | Taicang xinmaoyu 2 | Suzhou Taicang, Jiangsu | native breed | multi-cormel | spherosome | yellowish white | prolate ellipsoi | yellowish white | 77.35 | 429.28 | 506.63 | 5.55 |
| v29 | Yangyu 1 | Yangzhou, Jiangsu | bred varieties | multi-cormel | spherosome | light red | turbinate | light red | 25.29 | 235.20 | 260.49 | 9.30 |
| v30 | Yangyu 2 (green) | Yangzhou, Jiangsu | bred varieties | multi-cormel | spherosome | light red | ovoid | yellowish white | 76.07 | 411.45 | 487.52 | 5.41 |
| v31 | Yangyu 2 (red) | Yangzhou, Jiangsu | bred varieties | multi-cormel | spherosome | yellowish white | ovoid | yellowish white | 54.79 | 418.89 | 473.68 | 7.65 |
| v32 | Yongding lvyuehong | Longyan Yongding, Fujian | native breed | multi-cormel | spherosome | yellowish white | prolate ellipsoi | yellowish white | 47.81 | 361.24 | 409.05 | 7.56 |
| v34 | Xiaomianyu | Linyi, Shandong | native breed | multi-cormel | spherosome | light red | prolate ellipsoi | light red | 56.22 | 331.70 | 387.92 | 5.90 |
| v35 | Luyu 1 | Linyi, Shandong | native breed | multi-cormel | spherosome | yellowish white | ovoid | yellowish white | 52.82 | 320.50 | 373.32 | 6.07 |
| v36 | Luyu 3 | Linyi, Shandong | native breed | multi-cormel | spherosome | yellowish white | turbinate | yellowish white | 59.12 | 388.99 | 448.11 | 6.58 |
| v37 | Ribenhong | Taizhou, Jiangsu | native breed | multi-cormel | spherosome | yellowish white | ovoid | yellowish white | 103.78 | 451.45 | 555.23 | 4.35 |
| v38 | Diangyu 1 | Yuxi, Yunnan | native breed | single corm | spherosome | yellowish white | ovoid | yellowish white | 84.09 | 539.74 | 623.83 | 6.42 |
| v39 | Diangyu 4 | Yuxi, Yunnan | native breed | multi-cormel | spherosome | yellowish white | turbinate | yellowish white | 80.09 | 523.74 | 603.83 | 6.54 |
| v40 | Diangyu 8 | Yuxi, Yunnan | native breed | multi-cormel | flat and multi-head | light red | ovoid | light red | 65.68 | 403.13 | 468.81 | 6.14 |
| v41 | Eyu 1 | Wuhan, Hubei | native breed | multi-cormel | spherosome | light red | ovoid | yellowish white | 43.48 | 313.60 | 357.08 | 7.21 |
| v42 | Baiheyu | Taizhou, Jiangsu | native breed | multi-cormel | spherosome | yellowish white | prolate ellipsoi | yellowish white | 52.27 | 334.34 | 386.61 | 6.40 |
| v43 | Gouzhuayu | Qujing, Yunnan | native breed | multi-corm | spherosome | yellowish white | turbinate | yellowish white | 86.99 | 421.14 | 508.13 | 4.84 |
| v44 | Longyou hongyayu | Quzhou Longyou, Zhejiang | native breed | multi-cormel | spherosome | light red | prolate ellipsoi | light red | 89.19 | 456.51 | 545.70 | 5.12 |
| v45 | Quzhou yaowangyu | Quzhou, Zhejiang | native breed | multi-cormel | spherosome | light red | turbinate | light red | 79.47 | 439.05 | 518.52 | 5.52 |
| v46 | Fenghua yunai | Ningbo Fenghua, Zhejiang | native breed | single corm | spherosome | light red | prolate ellipsoi | light red | 65.97 | 387.24 | 453.21 | 5.87 |
| v47 | Fenghua yunai 2 | Ningbo Fenghua, Zhejiang | native breed | single corm | spherosome | yellowish white | prolate ellipsoi | yellowish white | 46.98 | 362.63 | 409.61 | 7.72 |
| v48 | Taiyu 2 | Taizhou Xinghua, Jiangsu | bred varieties | single corm | flat and multi-head | light red | prolate ellipsoi | light red | 121.00 | 490.52 | 611.51 | 4.05 |
| v49 | Xinghua longxiangyu | Taizhou Xinghua, Jiangsu | native breed | single corm | flat and multi-head | light red | ovoid | light red | 69.79 | 435.57 | 505.36 | 6.24 |
| v50 | Tengchong xianggengyu 1 | Tengchong, Yunnan | native breed | multi-corm | spherosome | yellowish white | turbinate | yellowish white | 82.51 | 431.88 | 514.39 | 5.23 |
| v51 | Tengchong xianggengyu 2 | Tengchong Yunnan | native breed | multi-corm | prolate and multi-head | yellowish white | prolate ellipsoi | yellowish white | 68.60 | 412.04 | 480.64 | 6.01 |
| v52 | Lipuyu | Guilin Lipu, Guangxi | native breed | single corm | spherosome | red violet | prolate ellipsoi | red violet | 67.12 | 411.36 | 478.49 | 6.13 |
| v53 | Fujian jiuzhangyu | Dehua Jiuzhang, Fujian | native breed | multi-corm | spherosome | light red | prolate ellipsoi | light red | 44.71 | 294.25 | 338.96 | 6.58 |
| v54 | Changshu xiangjiangyu | Suzhou Changshu, Jiangsu | native breed | single corm | spherosome | yellowish white | ovoid | yellowish white | 80.34 | 414.95 | 495.29 | 5.16 |
| v55 | Vi | Longyan Yongding, Fujian | native breed | multi-cormel | spherosome | yellowish white | prolate ellipsoi | yellowish white | 46.65 | 224.00 | 270.65 | 4.80 |
| v56 | LZ | Longyan Yongding, Fujian | native breed | multi-cormel | spherosome | light red | turbinate | light red | 75.56 | 332.12 | 407.68 | 4.40 |
| v57 | L1 | Longyan Yongding, Fujian | native breed | multi-cormel | spherosome | light red | prolate ellipsoi | light red | 94.73 | 305.69 | 400.41 | 3.23 |
| v58 | GLRZ | Longyan Yongding, Fujian | native breed | multi-cormel | spherosome | light red | ovoid | light red | 75.99 | 288.68 | 364.67 | 3.80 |
| v60 | Lipuyu lvgeng | Guilin Lipu, Guangxi | native breed | single corm | spherosome | light red | ovoid | light red | 79.27 | 371.37 | 450.65 | 4.68 |
| v61 | Ziyutou | Yangzhou Yizheng, Jiangsu | native breed | multi-cormel | spherosome | light red | ovoid | light red | 67.92 | 323.46 | 391.39 | 4.76 |
| v62 | Siyang yutou | Suqian Siyang, Jiangsu | native breed | multi-cormel | spherosome | yellowish white | prolate ellipsoi | yellowish white | 59.36 | 302.81 | 362.17 | 5.10 |
| v64 | Xinyuan xiangziyu | Suzhou Changshu, Jiangsu | native breed | multi-cormel | spherosome | light red | prolate ellipsoi | light red | 65.66 | 292.94 | 358.60 | 4.46 |
| v68 | Shuiyu | Suzhou Changshu, Jiangsu | native breed | multi-cormel | spherosome | yellowish white | prolate ellipsoi | yellowish white | 58.20 | 325.67 | 383.87 | 5.60 |

| | | | | | | | | | | | | |
|---|---|---|---|---|---|---|---|---|---|---|---|---|
| **v69** | Xinghua yutou | Taizhou Xinghua, Jiangsu | native breed | multi-cormel | spherosome | yellowish white | ovoid | yellowish white | 56.97 | 339.45 | 396.42 | 5.96 |
| **v70** | Ziyu | Yancheng Funing, Jiangsu | native breed | multi-cormel | spherosome | yellowish white | prolate ellipsoi | yellowish white | 47.60 | 316.23 | 363.83 | 6.64 |
| **v71** | Mashan xianggengyu | Wuxi Binhe, Jiangsu | native breed | multi-cormel | spherosome | yellowish white | prolate ellipsoi | light red | 97.58 | 415.24 | 512.82 | 4.26 |
| **v72** | Qinggengyu | Suzhou Taicang, Jiangsu | native breed | multi-cormel | spherosome | yellowish white | ovoid | yellowish white | 43.13 | 279.48 | 322.61 | 6.48 |
| **v73** | Qidong xiangshayu | Nantong Qidong, Jiangsu | native breed | multi-cormel | spherosome | light red | ovoid | light red | 95.85 | 356.86 | 452.71 | 3.72 |
| **v74** | yutou | Nantong Rudong, Jiangsu | native breed | multi-cormel | spherosome | yellowish white | ovoid | yellowish white | 54.19 | 316.47 | 370.65 | 5.84 |
| **v75** | Xiaoyutou | Suzhou Zhangjiagang, Jiangsu | native breed | multi-cormel | spherosome | red violet | prolate ellipsoi | red violet | 79.53 | 367.93 | 447.46 | 4.63 |
| **v76** | Maoyutou | Xuzhou Fengxian, Jiangsu | native breed | multi-cormel | spherosome | yellowish white | prolate ellipsoi | yellowish white | 59.52 | 338.31 | 397.83 | 5.68 |
| **v801** | Jinhua xiangyu | Jinhua Yongkang, Zhejiang | native breed | multi-cormel | spherosome | light red | ovoid | light red | 89.51 | 406.49 | 496.00 | 4.54 |
| **v802** | Xinyu hongyayu | Xinyu Yushui, Jiangxi | native breed | multi-cormel | spherosome | light red | prolate ellipsoi | light red | 95.03 | 432.45 | 527.47 | 4.55 |
| **v803** | Shangrao maoyutou | Shangrao Yushan, Jiangxi | native breed | multi-cormel | spherosome | light red | prolate ellipsoi | light red | 68.57 | 363.83 | 432.41 | 5.31 |
| **v804** | Zaozhuang maoyutou | Zaozhuang, Shandong | native breed | multi-cormel | spherosome | yellowish white | ovoid | yellowish white | 70.36 | 398.71 | 469.07 | 5.67 |
| **v805** | Chengmai xiaoyutou | Chengmai, Hainan | native breed | single corm | falt and multi-head | red violet | turbinate | red violet | 78.06 | 445.97 | 524.03 | 5.71 |
| **v806** | Ningshan yutou | Ningshan, Shaanxi | native breed | multi-cormel | spherosome | yellowish white | turbinate | yellowish white | 69.88 | 391.26 | 461.14 | 5.60 |
| **v807** | Meishan xiaomaoyu | Meishan Renshou, Sichuan | native breed | multi-cormel | spherosome | yellowish white | turbinate | yellowish white | 55.23 | 364.76 | 419.99 | 6.60 |
| **v809** | Linyi xiaoxiangyu | Linyi, Shandong | native breed | multi-cormel | spherosome | yellowish white | prolate ellipsoi | yellowish white | 58.50 | 365.27 | 423.77 | 6.24 |
| **v810** | Chuxiong yutou | Chuxiong, Yunnan | native breed | multi-cormel | spherosome | yellowish white with g | turbinate | yellowish white | 55.56 | 288.89 | 344.45 | 5.20 |
| **v811** | Miyun xiaomaoyutou | Miyun, Beijing | native breed | multi-cormel | spherosome | yellowish white | prolate ellipsoi | yellowish white | 54.52 | 253.26 | 307.78 | 4.65 |
| **v812** | Fenghua 1 | Ningbo Fenghua, Zhejiang | native breed | single corm | spherosome | red violet | ovoid | red violet | 79.10 | 434.18 | 513.28 | 5.49 |
| **v813** | Fenghua 2 | Ningbo Fenghua, Zhejiang | native breed | single corm | spherosome | red violet | ovoid | red violet | 203.86 | 225.53 | 429.39 | 1.11 |
| **v814** | Fenghua 3 | Ningbo Fenghua, Zhejiang | native breed | single corm | spherosome | yellowish white | ovoid | light red | 52.00 | 358.34 | 410.34 | 6.89 |
| **v815** | Kunming yutou | Kunming, Yunnan | native breed | multi-cormel | spherosome | red violet | prolate ellipsoi | red violet | 67.17 | 412.85 | 480.02 | 6.15 |
| **v816** | Xishuangbanna zigengyu | Xishuangbanna, Yunnan | native breed | single corm | spherosome | red violet | turbinate | red violet | 34.55 | 295.38 | 329.93 | 8.55 |
| **v817** | Xishuangbanna lvgengyu | Xishuangbanna, Yunnan | native breed | multi-cormel | spherosome | light red | turbinate | light red | 38.50 | 295.97 | 334.47 | 7.69 |
| **v818** | Xishuangbanna hongyayu | Xishuangbanna, Yunnan | bred varieties | single corm | turbinate | red violet | ovoid | red violet | 102.46 | 434.84 | 537.30 | 4.24 |
| **v819** | Xishuangbanna lvyagyu | Xishuangbanna, Yunnan | native breed | multi-cormel | prolate and multi-head | yellowish white with g | prolate ellipsoi | yellowish white | 73.31 | 414.35 | 487.66 | 5.65 |
| **v821** | Wangen | Yuxi, Yunnan | native breed | petiole type | spherosome | yellowish white | spherosome | yellowish white | 49.18 | 339.11 | 388.28 | 6.90 |
| **v822** | Laiyang lvyayu | Qingdao Laiyang, Shandong | native breed | multi-cormel | turbinate | yellowish white | ovoid | yellowish white | 65.45 | 315.11 | 380.56 | 4.81 |
| **v823** | Foshan yutou | Foshan, Guangdong | native breed | multi-cormel | ovoid | light red | prolate ellipsoi | light red | 100.52 | 387.24 | 487.76 | 3.85 |
| **v825** | Nanchang ziyuan 1 | Nanchang, Jiangxi | bred varieties | single corm | ovoid | yellowish white | turbinate | yellowish white | 158.15 | 135.28 | 293.43 | 0.86 |
| **v826** | Nanchang ziyuan 2 | Nanchang, Jiangxi | bred varieties | single corm | ovoid | yellowish white | turbinate | yellowish white | 156.47 | 225.35 | 381.82 | 1.44 |
| **v828** | Chongming xiangsuyu | Chongming, Shanghai | native breed | multi-cormel | spherosome | light red | ovoid | light red | 198.68 | 315.26 | 513.94 | 1.59 |
| **v843** | Qiantian yeyu | Qingtian, Zhejiang | native breed | multi-cormel | spherosome | light red | ovoid | light red | 83.94 | 440.94 | 524.88 | 5.25 |
