# Supplementary material for: Analysis of Gene Regulatory Networks of Taro (Colocasia esculenta (L.) Schott.) Soluble Starch Synthase Based on DeGN and KASP Marker Development
Source: Int J Genomics. 2025 Mar 1;2025:9953367. doi: 10.1155/ijog/9953367 (PMC11991784; doi:10.1155/ijog/9953367)
Supplement: Supporting Information — Additional supporting information can be found online in the Supporting Information section. Table S1: List of 89 taro resources, original source, variety type, morphotype, phenotypic traits, and starch component contents. Table S2: All transcript expression value (FPKM) in taro corm developing stage T1 to T6. Table S3: Gene interactions between DEGs in taro corm developing stage. Table S4: Expression profile of CeSSI, CeSS II, CeMy108, and SerThr kinase. Table S5: The genotypes of 89 taro resources. Table S6: 159 node genes of CeSS regulatory network. [file 9953367.f1.zip › Supplementary Table S3. Gene interactions between DEGs in taro cormel developing stage. (1).pdf]

Supplementary Table S3. Gene interactions between DEGs in taro cormel developing stage.

Source gene	Target gene	Degree of connection
EVM0000811	EVM0020628	1
EVM0016563	EVM0008570	0.991744
Colocasia_esculenta_newGene_771	EVM0001096	0.987616
EVM0008474	EVM0028456	0.985552
EVM0019373	EVM0020012	0.983488
EVM0014895	EVM0012409	0.981424
EVM0016224	EVM0020564	0.977296
EVM0016563	EVM0005180	0.977296
EVM0010536	EVM0020564	0.973168
EVM0007391	EVM0008474	0.973168
EVM0008570	EVM0005180	0.973168
EVM0022436	EVM0024069	0.973168
EVM0025628	EVM0016596	0.973168
EVM0012695	EVM0016010	0.971104
EVM0012409	EVM0010982	0.971104
EVM0004594	EVM0001733	0.971104
EVM0022715	EVM0020564	0.971104
EVM0015270	EVM0018411	0.971104
Colocasia_esculenta_newGene_901	EVM0020748	0.96904
EVM0018411	EVM0013713	0.96904
EVM0016224	EVM0022715	0.96904
EVM0021407	EVM0012409	0.96904
EVM0024682	EVM0024069	0.96904
EVM0016224	EVM0010536	0.96904
EVM0026948	EVM0024682	0.96904
EVM0020381	EVM0002433	0.966976
Colocasia_esculenta_newGene_956	EVM0004228	0.966976
EVM0018411	EVM0020748	0.966976
EVM0002433	EVM0001739	0.966976
EVM0020748	EVM0011473	0.964912
EVM0014597	EVM0023082	0.964912
EVM0021407	EVM0016596	0.964912
EVM0013713	EVM0020748	0.964912
EVM0012316	EVM0012650	0.964912
EVM0020564	EVM0024069	0.964912
EVM0028456	EVM0002082	0.962848
EVM0021407	EVM0009554	0.962848
EVM0007391	EVM0015270	0.962848
EVM0022715	EVM0010536	0.962848
EVM0002433	EVM0018643	0.962848
EVM0007391	EVM0016224	0.962848
EVM0008474	EVM0002433	0.962848
EVM0022436	EVM0024682	0.962848
EVM0010332	EVM0002082	0.962848
EVM0001853	EVM0020564	0.962848
EVM0016728	EVM0013500	0.960784
EVM0008210	Colocasia_escule	0.960784
EVM0027449	EVM0013713	0.960784
EVM0022715	EVM0013669	0.960784
EVM0022715	EVM0007532	0.960784
EVM0020564	EVM0008570	0.960784
EVM0022715	EVM0014369	0.960784
EVM0014597	EVM0012607	0.960784
EVM0021407	EVM0010982	0.960784
EVM0026948	EVM0018701	0.960784
EVM0020564	EVM0002433	0.95872

EVM0016224	EVM0022133	0.95872
EVM0007391	EVM0002433	0.95872
EVM0005345	EVM0010905	0.95872
EVM0007214	EVM0021380	0.95872
EVM0027449	EVM0020748	0.95872
EVM0012607	EVM0010982	0.95872
EVM0010332	EVM0022133	0.95872
EVM0022715	EVM0009322	0.95872
EVM0018643	EVM0007532	0.95872
EVM0015270	EVM0013713	0.95872
EVM0020381	EVM0023059	0.956656
EVM0012316	EVM0013500	0.956656
EVM0016010	EVM0010982	0.956656
Colocasia_esculenta_newGene_863	EVM0000765	0.956656
EVM0009322	EVM0024069	0.956656
EVM0003916	EVM0010982	0.956656
EVM0007391	EVM0018411	0.956656
EVM0000765	EVM0028138	0.956656
EVM0002433	EVM0012655	0.956656
Colocasia_esculenta_newGene_901	EVM0011632	0.956656
EVM0014597	EVM0008210	0.956656
Colocasia_esculenta_newGene_901	EVM0011473	0.956656
EVM0000541	EVM0012650	0.956656
Colocasia_esculenta_newGene_865	EVM0023938	0.956656
EVM0019373	EVM0000541	0.956656
Colocasia_esculenta_newGene_865	EVM0012655	0.956656
EVM0010086	EVM0020012	0.956656
EVM0005345	EVM0021407	0.956656
EVM0026948	EVM0001947	0.956656
EVM0008210	EVM0005180	0.954592
EVM0021390	EVM0026177	0.954592
EVM0016224	EVM0008474	0.954592
Colocasia_esculenta_newGene_863	EVM0003916	0.954592
EVM0022133	EVM0006551	0.954592
EVM0014597	EVM0005345	0.954592
EVM0008474	EVM0015270	0.954592
EVM0022715	EVM0002433	0.954592
EVM0012316	EVM0020012	0.954592
EVM0026948	EVM0022436	0.954592
EVM0005432	EVM0014706	0.954592
EVM0026948	EVM0010332	0.954592
Colocasia_esculenta_newGene_863	EVM0012409	0.954592
EVM0023082	EVM0010905	0.954592
EVM0008210	EVM0000541	0.954592
EVM0009174	EVM0007856	0.954592
EVM0016194	EVM0018490	0.954592
EVM0010332	EVM0000541	0.952528
EVM0007391	EVM0020564	0.952528
EVM0007214	EVM0011820	0.952528
EVM0005345	EVM0010982	0.952528
EVM0009322	EVM0007532	0.952528
EVM0016606	EVM0002082	0.952528
EVM0016563	EVM0002433	0.952528
EVM0016596	EVM0012409	0.952528
EVM0007391	EVM0001739	0.952528
EVM0025628	EVM0021407	0.952528
EVM0028456	EVM0002433	0.952528
EVM0012695	EVM0012409	0.952528

EVM0021407	EVM0014895	0.952528
EVM0013713	EVM0006551	0.952528
EVM0023059	EVM0024069	0.952528
EVM0008210	EVM0012650	0.952528
EVM0016224	EVM0013669	0.952528
EVM0008210	EVM0012409	0.952528
EVM0001853	EVM0004228	0.952528
EVM0005345	EVM0012409	0.952528
EVM0016194	EVM0001096	0.952528
EVM0007391	EVM0027449	0.952528
EVM0012650	EVM0028206	0.952528
EVM0012409	EVM0002524	0.952528
EVM0010332	EVM0023576	0.950464
EVM0023059	EVM0002433	0.950464
EVM0007391	EVM0013713	0.950464
EVM0016224	EVM0001853	0.950464
EVM0008948	EVM0022327	0.950464
EVM0015080	EVM0016156	0.950464
EVM0021407	EVM0010905	0.950464
EVM0000541	EVM0013500	0.950464
EVM0008210	EVM0002433	0.950464
Colocasia_esculenta_newGene_863	EVM0015541	0.950464
EVM0016596	EVM0014895	0.950464
EVM0028456	EVM0024069	0.950464
EVM0002433	EVM0005180	0.950464
EVM0000541	EVM0014369	0.950464
EVM0007214	EVM0000765	0.950464
EVM0008210	EVM0019373	0.950464
EVM0026948	EVM0008474	0.950464
EVM0008474	EVM0020564	0.950464
EVM0012316	EVM0019373	0.950464
EVM0009554	EVM0007090	0.9484
EVM0028456	EVM0024682	0.9484
EVM0016563	EVM0008602	0.9484
EVM0020012	EVM0023082	0.9484
EVM0022133	EVM0005432	0.9484
EVM0025980	Colocasia_escule	0.9484
EVM0019373	EVM0020593	0.9484
EVM0008474	EVM0024069	0.9484
EVM0008210	EVM0003916	0.9484
EVM0012409	EVM0003916	0.9484
EVM0024682	EVM0014706	0.9484
EVM0026948	EVM0022715	0.9484
EVM0022715	EVM0006304	0.9484
EVM0016606	EVM0023576	0.9484
EVM0010905	EVM0018490	0.9484
EVM0014895	EVM0002524	0.9484
EVM0025117	Colocasia_escule	0.9484
EVM0010086	EVM0020593	0.9484
EVM0026948	EVM0018411	0.9484
EVM0001224	EVM0023403	0.9484
EVM0020564	EVM0027189	0.9484
EVM0012695	Colocasia_escule	0.9484
EVM0020012	EVM0020593	0.9484
EVM0014895	Colocasia_escule	0.9484
EVM0019373	EVM0023082	0.9484
EVM0023970	EVM0006551	0.946336
EVM0015080	EVM0028138	0.946336

EVM0008474	EVM0002082	0.946336
EVM0014597	EVM0010982	0.946336
EVM0008602	EVM0019956	0.946336
EVM0016224	EVM0002433	0.946336
EVM0026948	EVM0008519	0.946336
EVM0022715	EVM0000541	0.946336
Colocasia_esculenta_newGene_771	EVM0026115	0.946336
EVM0012607	EVM0026115	0.946336
EVM0010332	EVM0008519	0.946336
EVM0007391	Colocasia_escule	0.946336
EVM0000541	EVM0026358	0.946336
EVM0008474	EVM0018411	0.946336
Colocasia_esculenta_newGene_783	EVM0028206	0.946336
EVM0004594	EVM0021380	0.946336
EVM0004594	EVM0025803	0.946336
EVM0015541	EVM0000765	0.946336
EVM0015270	EVM0006551	0.946336
EVM0000541	EVM0002433	0.946336
EVM0010536	EVM0013669	0.946336
EVM0012607	EVM0021407	0.946336
EVM0002433	EVM0008570	0.946336
EVM0016224	EVM0026948	0.946336
EVM0005345	EVM0003916	0.946336
EVM0020012	EVM0028206	0.946336
EVM0026948	EVM0024069	0.946336
EVM0016194	EVM0009554	0.946336
EVM0008210	EVM0010982	0.946336
EVM0014597	EVM0021407	0.946336
EVM0016563	EVM0020564	0.946336
EVM0013823	EVM0025117	0.946336
EVM0012607	EVM0008210	0.946336
Colocasia_esculenta_newGene_956	EVM0012655	0.944272
EVM0020564	EVM0017284	0.944272
EVM0023082	EVM0020593	0.944272
EVM0014597	EVM0012409	0.944272
EVM0010530	EVM0022436	0.944272
EVM0012695	EVM0010982	0.944272
EVM0026948	EVM0023970	0.944272
EVM0010332	EVM0022436	0.944272
EVM0008602	EVM0008570	0.944272
EVM0021407	EVM0003916	0.944272
EVM0012607	EVM0012409	0.944272
EVM0002590	EVM0015709	0.944272
EVM0023059	EVM0016606	0.944272
EVM0008474	EVM0020748	0.944272
EVM0002433	EVM0002082	0.944272
EVM0002433	EVM0012650	0.944272
EVM0007391	EVM0010086	0.944272
EVM0002590	EVM0005432	0.944272
EVM0027553	EVM0028456	0.944272
EVM0007391	EVM0010332	0.944272
Colocasia_esculenta_newGene_901	EVM0018701	0.944272
EVM0001853	EVM0024069	0.944272
EVM0008474	EVM0024682	0.944272
EVM0007391	EVM0002082	0.944272
EVM0007391	EVM0020748	0.944272
Colocasia_esculenta_newGene_783	EVM0002433	0.944272
EVM0010536	EVM0002433	0.944272

EVM0007391	EVM0028456	0.944272
EVM0010332	EVM0006551	0.944272
EVM0018411	EVM0006551	0.944272
Colocasia_esculenta_newGene_239	EVM0018490	0.944272
EVM0019373	EVM0013500	0.944272
EVM0002433	EVM0007532	0.944272
EVM0011632	EVM0020748	0.944272
EVM0012607	EVM0005345	0.942208
EVM0023059	EVM0017284	0.942208
EVM0020748	EVM0006551	0.942208
EVM0026948	EVM0015709	0.942208
EVM0016606	EVM0000541	0.942208
Colocasia_esculenta_newGene_647	EVM0006304	0.942208
EVM0005581	EVM0002082	0.942208
EVM0008210	EVM0018411	0.942208
EVM0012650	EVM0013500	0.942208
EVM0010982	Colocasia_escule	0.942208
EVM0014895	EVM0010982	0.942208
EVM0010332	EVM0020564	0.942208
EVM0007391	EVM0020593	0.942208
EVM0008210	EVM0012316	0.942208
EVM0021407	Colocasia_escule	0.942208
EVM0016008	EVM0009174	0.942208
EVM0015709	EVM0012655	0.942208
EVM0008372	EVM0009554	0.942208
Colocasia_esculenta_newGene_863	EVM0028138	0.942208
EVM0012607	EVM0026358	0.942208
EVM0014597	EVM0003916	0.942208
EVM0012607	EVM0003916	0.942208
EVM0018411	EVM0012650	0.942208
EVM0021380	EVM0000765	0.942208
EVM0007391	EVM0001853	0.942208
EVM0014597	EVM0018490	0.942208
EVM0021380	EVM0001733	0.942208
EVM0018411	EVM0007856	0.942208
EVM0016224	EVM0010332	0.942208
EVM0007391	EVM0005581	0.942208
EVM0023970	EVM0015709	0.942208
EVM0023918	EVM0005581	0.942208
EVM0014597	EVM0005180	0.942208
EVM0019373	EVM0002433	0.942208
EVM0028456	EVM0023576	0.942208
EVM0015270	EVM0003916	0.942208
EVM0015270	Colocasia_escule	0.942208
EVM0022715	EVM0024069	0.942208
EVM0005581	EVM0013713	0.942208
EVM0016606	EVM0028456	0.942208
EVM0005345	EVM0000767	0.940144
EVM0008210	EVM0020012	0.940144
EVM0022436	EVM0009322	0.940144
EVM0022133	EVM0020564	0.940144
EVM0014597	EVM0006304	0.940144
EVM0021407	EVM0018490	0.940144
EVM0010332	EVM0028456	0.940144
EVM0027935	EVM0002433	0.940144
EVM0020216	EVM0010536	0.940144
EVM0010332	EVM0015270	0.940144
EVM0006304	EVM0000541	0.940144

EVM0018490	EVM0001096	0.940144
EVM0027715	EVM0000811	0.940144
EVM0015080	EVM0012409	0.940144
EVM0005581	Colocasia_escule	0.940144
EVM0014895	EVM0012695	0.940144
EVM0010332	EVM0001947	0.940144
Colocasia_esculenta_newGene_462	EVM0018411	0.940144
EVM0023918	EVM0007649	0.940144
EVM0002082	EVM0001739	0.940144
EVM0010332	EVM0023970	0.940144
EVM0024474	EVM0018760	0.940144
EVM0012316	EVM0000541	0.940144
EVM0010332	EVM0016606	0.940144
EVM0014895	EVM0011820	0.940144
EVM0027935	EVM0022327	0.940144
EVM0027449	EVM0011473	0.940144
EVM0001224	EVM0016606	0.940144
EVM0023082	EVM0018490	0.940144
EVM0016224	EVM0013713	0.940144
EVM0010536	EVM0001853	0.940144
EVM0012409	EVM0000767	0.940144
EVM0008372	EVM0012360	0.940144
EVM0007391	EVM0022715	0.940144
EVM0020564	EVM0028456	0.940144
EVM0014895	EVM0003916	0.940144
EVM0020564	EVM0027734	0.940144
Colocasia_esculenta_newGene_647	EVM0014369	0.940144
EVM0025117	EVM0005844	0.940144
EVM0027734	EVM0017284	0.940144
EVM0016224	EVM0005432	0.940144
EVM0008210	EVM0015270	0.940144
EVM0016224	EVM0024069	0.940144
EVM0020564	EVM0024682	0.940144
EVM0027715	EVM0020628	0.940144
EVM0009554	EVM0012360	0.940144
EVM0020564	EVM0023576	0.93808
EVM0008474	EVM0001739	0.93808
EVM0015709	EVM0000767	0.93808
EVM0016224	EVM0024682	0.93808
EVM0025117	EVM0001733	0.93808
EVM0020216	EVM0006551	0.93808
EVM0022436	EVM0007856	0.93808
EVM0016224	EVM0001947	0.93808
EVM0008210	EVM0015541	0.93808
EVM0021407	EVM0007090	0.93808
EVM0006304	EVM0020564	0.93808
EVM0024682	EVM0018701	0.93808
EVM0020564	EVM0009322	0.93808
EVM0020564	EVM0020748	0.93808
EVM0001281	EVM0020564	0.93808
EVM0010332	EVM0003916	0.93808
EVM0014597	EVM0022327	0.93808
EVM0020012	EVM0013500	0.93808
EVM0010332	EVM0024069	0.93808
EVM0008474	EVM0010332	0.93808
EVM0008474	EVM0022715	0.93808
EVM0010086	EVM0013713	0.93808
EVM0016596	EVM0010982	0.93808

Colocasia_esculenta_newGene_863	EVM0016010	0.93808
EVM0001224	EVM0023576	0.93808
EVM0010086	EVM0015270	0.93808
EVM0002433	EVM0020593	0.93808
Colocasia_esculenta_newGene_865	EVM0002433	0.93808
EVM0010332	EVM0003656	0.93808
EVM0014895	EVM0020012	0.93808
EVM0008474	EVM0013713	0.93808
EVM0025628	Colocasia_escule	0.93808
EVM0014597	EVM0014895	0.93808
EVM0007391	EVM0026948	0.93808
EVM0003916	EVM0005844	0.93808
EVM0020564	EVM0004228	0.93808
EVM0002433	EVM0024069	0.93808
EVM0009554	EVM0008519	0.93808
EVM0016606	EVM0013500	0.93808
EVM0027734	EVM0023576	0.93808
EVM0016224	EVM0018411	0.93808
EVM0026948	EVM0020564	0.93808
EVM0012607	EVM0018411	0.93808
EVM0022715	EVM0001853	0.93808
EVM0026948	EVM0003916	0.93808
EVM0022715	EVM0001947	0.93808
EVM0005432	EVM0027189	0.93808
EVM0005432	EVM0006551	0.93808
EVM0026948	EVM0002590	0.93808
EVM0020564	EVM0007532	0.93808
EVM0020381	EVM0018643	0.93808
EVM0026948	EVM0028456	0.93808
EVM0008210	EVM0016563	0.93808
EVM0012316	EVM0028206	0.93808
EVM0009554	EVM0018490	0.936017
EVM0012695	EVM0015541	0.936017
EVM0018411	EVM0013669	0.936017
EVM0024474	EVM0009322	0.936017
EVM0012607	EVM0015709	0.936017
EVM0020564	EVM0000541	0.936017
EVM0023059	EVM0028456	0.936017
EVM0022715	EVM0027734	0.936017
Colocasia_esculenta_newGene_863	EVM0005180	0.936017
EVM0001281	EVM0027189	0.936017
EVM0012607	EVM0000541	0.936017
EVM0006304	EVM0008570	0.936017
EVM0014597	Colocasia_escule	0.936017
EVM0012607	EVM0015270	0.936017
EVM0020012	EVM0012409	0.936017
EVM0018411	EVM0015709	0.936017
EVM0021407	EVM0015270	0.936017
EVM0010332	EVM0001224	0.936017
EVM0018701	EVM0011473	0.936017
EVM0010086	EVM0028206	0.936017
Colocasia_esculenta_newGene_462	EVM0015709	0.936017
EVM0015270	EVM0012409	0.936017
EVM0022133	EVM0027189	0.936017
EVM0010536	EVM0018643	0.936017
EVM0005432	EVM0000767	0.936017
EVM0020564	EVM0019956	0.936017
EVM0022436	EVM0000541	0.936017

EVM0022715	EVM0010332	0.936017
EVM0012409	EVM0022327	0.936017
EVM0013713	EVM0011473	0.936017
EVM0017284	EVM0024069	0.936017
EVM0011820	EVM0003916	0.936017
EVM0005432	EVM0020564	0.936017
EVM0008210	EVM0028138	0.936017
EVM0002590	EVM0012655	0.936017
EVM0022715	EVM0024474	0.936017
EVM0010332	EVM0018411	0.936017
EVM0005432	EVM0015709	0.936017
EVM0016596	EVM0022327	0.936017
EVM0016010	EVM0003916	0.936017
EVM0005432	EVM0024682	0.936017
EVM0012607	EVM0020308	0.936017
EVM0005581	EVM0027449	0.936017
EVM0027935	EVM0020593	0.936017
EVM0006304	EVM0002433	0.936017
EVM0009174	EVM0020748	0.936017
EVM0025628	EVM0012409	0.936017
EVM0018760	EVM0014706	0.936017
EVM0010332	EVM0013713	0.936017
EVM0023576	EVM0001739	0.936017
EVM0012409	EVM0015541	0.936017
EVM0022133	EVM0013713	0.936017
EVM0012607	Colocasia_escule	0.936017
EVM0023938	EVM0007649	0.936017
EVM0012607	EVM0002433	0.933953
EVM0023918	EVM0007391	0.933953
EVM0016224	EVM0015270	0.933953
EVM0006304	EVM0016563	0.933953
Colocasia_esculenta_newGene_863	EVM0010982	0.933953
Colocasia_esculenta_newGene_865	EVM0020381	0.933953
EVM0009554	EVM0003916	0.933953
Colocasia_esculenta_newGene_901	EVM0024069	0.933953
EVM0008210	EVM0016010	0.933953
EVM0018643	EVM0001739	0.933953
EVM0016563	EVM0012655	0.933953
EVM0018643	EVM0007856	0.933953
EVM0002433	EVM0017284	0.933953
EVM0028456	EVM0001739	0.933953
EVM0012650	EVM0007856	0.933953
EVM0007391	EVM0024069	0.933953
EVM0014597	EVM0010905	0.933953
EVM0014597	EVM0008948	0.933953
EVM0014597	EVM0011820	0.933953
EVM0002433	EVM0007856	0.933953
EVM0013713	EVM0009174	0.933953
EVM0012409	EVM0028138	0.933953
Colocasia_esculenta_newGene_462	EVM0010905	0.933953
EVM0022436	EVM0003916	0.933953
EVM0018411	EVM0011473	0.933953
EVM0026948	EVM0000541	0.933953
EVM0009322	EVM0007856	0.933953
EVM0020748	EVM0018701	0.933953
EVM0022436	EVM0020748	0.933953
Colocasia_esculenta_newGene_239	Colocasia_escule	0.933953
EVM0027734	EVM0020748	0.933953

Colocasia_esculenta_newGene_771	EVM0018490	0.933953
EVM0007214	EVM0008210	0.933953
EVM0010536	EVM0004228	0.933953
EVM0020593	EVM0028206	0.933953
EVM0018411	EVM0009174	0.933953
EVM0010536	EVM0008570	0.933953
EVM0016606	EVM0002433	0.933953
EVM0016563	EVM0004228	0.933953
EVM0010536	EVM0027734	0.933953
EVM0022715	Colocasia_escule	0.933953
EVM0022715	EVM0022436	0.933953
EVM0020564	EVM0005180	0.933953
EVM0002082	EVM0008519	0.933953
EVM0023938	EVM0002433	0.933953
EVM0007391	EVM0017284	0.933953
EVM0004228	EVM0008570	0.933953
EVM0007649	EVM0020012	0.933953
EVM0004228	EVM0005180	0.933953
EVM0015080	EVM0014895	0.933953
EVM0010905	EVM0015709	0.933953
EVM0008210	EVM0000765	0.933953
EVM0023918	EVM0020216	0.933953
EVM0007391	EVM0018643	0.933953
EVM0008570	EVM0024069	0.933953
EVM0022133	EVM0002082	0.933953
EVM0023576	EVM0002082	0.933953
EVM0008210	EVM0014895	0.933953
EVM0024682	EVM0000767	0.933953
EVM0024682	EVM0015709	0.933953
EVM0026948	EVM0024474	0.933953
EVM0001739	EVM0007532	0.931889
EVM0023082	EVM0008519	0.931889
EVM0008474	EVM0027734	0.931889
EVM0019373	EVM0015270	0.931889
EVM0021848	EVM0005844	0.931889
Colocasia_esculenta_newGene_462	EVM0013713	0.931889
Colocasia_esculenta_newGene_863	EVM0018701	0.931889
EVM0018760	EVM0011473	0.931889
EVM0018490	EVM0008519	0.931889
EVM0022715	EVM0018643	0.931889
EVM0014895	EVM0028138	0.931889
EVM0002433	EVM0004228	0.931889
EVM0008210	EVM0011820	0.931889
EVM0004228	EVM0012655	0.931889
EVM0015080	EVM0025117	0.931889
EVM0027407	EVM0018701	0.931889
EVM0021407	EVM0023082	0.931889
EVM0012607	Colocasia_escule	0.931889
EVM0005581	EVM0002433	0.931889
EVM0012607	EVM0023059	0.931889
EVM0005581	EVM0016606	0.931889
EVM0008210	EVM0020564	0.931889
EVM0016224	EVM0002082	0.931889
EVM0020593	EVM0001739	0.931889
EVM0013047	EVM0027189	0.931889
EVM0021407	EVM0022327	0.931889
EVM0023059	EVM0022436	0.931889
EVM0026948	EVM0014369	0.931889

EVM0023938	EVM0019373	0.931889
EVM0019309	EVM0022327	0.931889
EVM0011820	EVM0012409	0.931889
EVM0023403	EVM0002082	0.931889
EVM0026948	EVM0009322	0.931889
EVM0002590	EVM0004228	0.931889
EVM0002590	EVM0024682	0.931889
EVM0005345	EVM0023082	0.931889
EVM0005345	EVM0009554	0.931889
EVM0014597	EVM0002433	0.931889
EVM0016596	EVM0012695	0.931889
EVM0022715	EVM0016563	0.931889
EVM0007391	Colocasia_escule	0.931889
Colocasia_esculenta_newGene_901	EVM0020564	0.931889
EVM0018411	EVM0003916	0.931889
EVM0016224	Colocasia_escule	0.931889
EVM0019956	EVM0001739	0.931889
EVM0022715	EVM0001739	0.931889
EVM0001096	EVM0008519	0.931889
EVM0008948	EVM0010982	0.931889
EVM0020564	EVM0018643	0.931889
EVM0014597	EVM0000541	0.931889
EVM0012607	EVM0023082	0.931889
EVM0008474	EVM0007856	0.929825
EVM0025803	EVM0001733	0.929825
EVM0008474	EVM0018643	0.929825
EVM0022715	EVM0024682	0.929825
Colocasia_esculenta_newGene_647	EVM0000541	0.929825
EVM0010332	EVM0020012	0.929825
EVM0008474	EVM0011473	0.929825
EVM0008602	EVM0005180	0.929825
EVM0003916	EVM0000765	0.929825
EVM0018411	EVM0008519	0.929825
EVM0022715	EVM0018411	0.929825
EVM0018760	EVM0017550	0.929825
EVM0008210	EVM0022327	0.929825
EVM0008210	EVM0004228	0.929825
EVM0022715	EVM0008570	0.929825
EVM0022715	EVM0022133	0.929825
EVM0022715	EVM0019956	0.929825
EVM0006304	EVM0023082	0.929825
EVM0008210	EVM0008570	0.929825
EVM0012607	EVM0012650	0.929825
EVM0012607	EVM0005180	0.929825
EVM0018643	EVM0013500	0.929825
EVM0026948	EVM0005345	0.929825
EVM0025628	EVM0010982	0.929825
EVM0012316	EVM0020308	0.929825
EVM0000541	EVM0018411	0.929825
EVM0016596	EVM0009554	0.929825
EVM0026948	EVM0020748	0.929825
EVM0022436	EVM0014706	0.929825
EVM0017284	EVM0020748	0.929825
EVM0014369	EVM0009322	0.929825
EVM0009233	EVM0025980	0.929825
EVM0020748	EVM0007856	0.929825
EVM0014597	EVM0019373	0.929825
EVM0020748	EVM0024069	0.929825

EVM0020216	EVM0019229	0.929825
EVM0007214	EVM0015080	0.929825
EVM0016224	EVM0027189	0.929825
EVM0023059	EVM0000541	0.929825
EVM0014706	EVM0024069	0.929825
EVM0020012	EVM0019309	0.929825
EVM0025117	EVM0010982	0.929825
EVM0020564	EVM0013669	0.929825
EVM0011820	Colocasia_escule	0.929825
EVM0010536	EVM0027189	0.929825
EVM0016224	EVM0028456	0.929825
EVM0020381	Colocasia_escule	0.929825
EVM0011632	EVM0000767	0.929825
EVM0020012	EVM0000541	0.929825
EVM0008372	EVM0016194	0.929825
EVM0012607	EVM0018490	0.929825
EVM0020012	EVM0015270	0.929825
EVM0016224	EVM0008210	0.929825
EVM0012607	EVM0024682	0.929825
EVM0019373	EVM0019309	0.929825
EVM0015270	EVM0020748	0.929825
EVM0015270	EVM0008519	0.929825
Colocasia_esculenta_newGene_901	EVM0018411	0.929825
Colocasia_esculenta_newGene_901	EVM0013713	0.929825
EVM0018411	EVM0002433	0.929825
EVM0019373	EVM0000811	0.929825
EVM0024474	EVM0023082	0.929825
EVM0002433	EVM0024682	0.929825
EVM0014895	EVM0015541	0.929825
EVM0002082	EVM0024069	0.929825
EVM0010086	EVM0018411	0.929825
EVM0010086	EVM0003656	0.929825
EVM0016008	EVM0014895	0.929825
EVM0001739	EVM0008519	0.929825
EVM0010536	EVM0022133	0.929825
EVM0008474	EVM0008210	0.929825
EVM0008474	EVM0027553	0.929825
EVM0008474	EVM0022436	0.929825
EVM0019373	EVM0028206	0.929825
EVM0006551	EVM0011473	0.929825
EVM0020216	EVM0013713	0.929825
EVM0019373	EVM0012650	0.929825
EVM0019373	EVM0020628	0.929825
EVM0001281	EVM0005432	0.929825
EVM0010536	EVM0005432	0.927761
Colocasia_esculenta_newGene_239	EVM0015709	0.927761
Colocasia_esculenta_newGene_239	EVM0020628	0.927761
EVM0009588	EVM0022436	0.927761
EVM0014895	EVM0000765	0.927761
EVM0016224	EVM0018643	0.927761
EVM0009174	EVM0028206	0.927761
EVM0009588	EVM0014706	0.927761
EVM0020748	EVM0012650	0.927761
EVM0015270	EVM0002433	0.927761
EVM0003916	EVM0020308	0.927761
Colocasia_esculenta_newGene_956	EVM0002433	0.927761
EVM0001281	EVM0022133	0.927761
EVM0007649	EVM0015270	0.927761

Colocasia_esculenta_newGene_956	EVM0016563	0.927761
EVM0020564	EVM0018411	0.927761
EVM0020564	EVM0018760	0.927761
EVM0027734	EVM0002433	0.927761
EVM0016563	EVM0027935	0.927761
EVM0001281	EVM0001224	0.927761
EVM0014578	EVM0001733	0.927761
EVM0015270	EVM0010982	0.927761
EVM0023059	EVM0002082	0.927761
EVM0010536	EVM0016563	0.927761
EVM0012655	EVM0001739	0.927761
EVM0012409	EVM0006551	0.927761
EVM0016606	EVM0024069	0.927761
Colocasia_esculenta_newGene_239	EVM0000811	0.927761
EVM0018760	EVM0009322	0.927761
EVM0026948	EVM0015270	0.927761
EVM0008474	EVM0017284	0.927761
EVM0026948	Colocasia_escule	0.927761
EVM0015541	EVM0010982	0.927761
EVM0026948	EVM0018760	0.927761
EVM0023082	EVM0019309	0.927761
EVM0026948	EVM0012360	0.927761
EVM0002433	EVM0013500	0.927761
EVM0026948	EVM0002082	0.927761
EVM0008474	EVM0012650	0.927761
EVM0019229	EVM0027734	0.927761
EVM0019373	EVM0022327	0.927761
EVM0020593	EVM0013500	0.927761
EVM0009233	EVM0010982	0.927761
EVM0008474	EVM0005180	0.927761
EVM0028456	EVM0020748	0.927761
EVM0021407	EVM0012695	0.927761
EVM0019229	EVM0001224	0.927761
EVM0008474	EVM0018701	0.927761
EVM0016728	EVM0012650	0.927761
EVM0005581	EVM0020593	0.927761
EVM0024682	EVM0002082	0.927761
EVM0008474	EVM0010536	0.927761
EVM0024474	EVM0001947	0.927761
EVM0010332	EVM0020748	0.927761
EVM0007214	EVM0015541	0.927761
EVM0012607	EVM0026948	0.927761
EVM0006304	EVM0024474	0.927761
EVM0020381	EVM0007856	0.927761
EVM0020381	EVM0012655	0.927761
EVM0008474	EVM0004228	0.927761
EVM0022133	EVM0001947	0.927761
EVM0023576	EVM0024069	0.927761
EVM0019373	EVM0012409	0.927761
EVM0020564	EVM0001739	0.927761
Colocasia_esculenta_newGene_570	EVM0009174	0.927761
EVM0022133	EVM0023970	0.927761
EVM0008210	Colocasia_escule	0.927761
EVM0026948	Colocasia_escule	0.927761
EVM0027935	EVM0008570	0.927761
EVM0025803	EVM0024351	0.927761
EVM0027449	EVM0006551	0.927761
EVM0022436	EVM0018411	0.927761

EVM0014895	EVM0022327	0.925697
EVM0005345	EVM0015709	0.925697
EVM0006304	EVM0024069	0.925697
EVM0025117	Colocasia_escule	0.925697
EVM0025117	EVM0025980	0.925697
EVM0025117	EVM0012409	0.925697
EVM0003916	EVM0012360	0.925697
EVM0020381	EVM0017284	0.925697
EVM0006304	EVM0009322	0.925697
EVM0016606	EVM0020564	0.925697
EVM0009531	EVM0021848	0.925697
EVM0021390	EVM0012360	0.925697
EVM0021390	EVM0005844	0.925697
EVM0003916	EVM0012650	0.925697
EVM0000541	EVM0018643	0.925697
EVM0021407	EVM0008519	0.925697
EVM0007649	EVM0017284	0.925697
EVM0012409	EVM0000765	0.925697
EVM0000541	EVM0024069	0.925697
EVM0012409	EVM0008948	0.925697
EVM0000541	EVM0007532	0.925697
EVM0012409	EVM0010905	0.925697
EVM0016224	EVM0024474	0.925697
EVM0022436	EVM0020564	0.925697
EVM0023918	Colocasia_escule	0.925697
EVM0015270	EVM0012650	0.925697
EVM0001281	EVM0016224	0.925697
EVM0007856	EVM0024069	0.925697
EVM0014597	EVM0009554	0.925697
EVM0015541	EVM0006897	0.925697
EVM0026948	EVM0023082	0.925697
EVM0013713	EVM0007856	0.925697
EVM0008210	EVM0021407	0.925697
EVM0026948	EVM0013669	0.925697
EVM0008210	EVM0022715	0.925697
EVM0022436	EVM0014369	0.925697
EVM0005432	Colocasia_escule	0.925697
EVM0015270	EVM0023970	0.925697
EVM0004228	EVM0024069	0.925697
EVM0014597	EVM0026948	0.925697
EVM0022436	EVM0002082	0.925697
EVM0008474	EVM0003916	0.925697
EVM0019229	EVM0008210	0.925697
EVM0022436	EVM0017284	0.925697
EVM0008474	Colocasia_escule	0.925697
EVM0014597	EVM0020012	0.925697
EVM0008474	EVM0023576	0.925697
EVM0002433	EVM0014369	0.925697
EVM0018760	EVM0024069	0.925697
EVM0023970	EVM0008519	0.925697
EVM0014578	EVM0025803	0.925697
EVM0023059	EVM0020564	0.925697
EVM0022715	EVM0018760	0.925697
EVM0026363	EVM0020308	0.925697
EVM0016224	EVM0006551	0.925697
EVM0026363	EVM0027715	0.925697
EVM0013669	EVM0001947	0.925697
EVM0016224	EVM0020748	0.925697

EVM0016224	EVM0018760	0.925697
EVM0014706	EVM0006551	0.925697
Colocasia_esculenta_newGene_901	EVM0018760	0.925697
EVM0001853	EVM0008570	0.925697
EVM0020012	EVM0015541	0.925697
EVM0008210	EVM0020308	0.925697
Colocasia_esculenta_newGene_462	EVM0000767	0.925697
EVM0008210	EVM0024682	0.925697
Colocasia_esculenta_newGene_901	EVM0008474	0.925697
EVM0019373	EVM0016156	0.923633
EVM0014597	EVM0024682	0.923633
EVM0007649	EVM0019373	0.923633
EVM0015270	EVM0000541	0.923633
EVM0001947	EVM0009322	0.923633
EVM0007391	EVM0012607	0.923633
EVM0010905	EVM0008519	0.923633
Colocasia_esculenta_newGene_863	EVM0005844	0.923633
EVM0027449	Colocasia_escule	0.923633
EVM0019373	EVM0018490	0.923633
EVM0019373	EVM0018643	0.923633
EVM0022133	EVM0001853	0.923633
Colocasia_esculenta_newGene_863	EVM0012650	0.923633
EVM0007649	EVM0027734	0.923633
EVM0007391	EVM0022133	0.923633
EVM0002433	EVM0019956	0.923633
EVM0007391	EVM0010536	0.923633
EVM0016563	EVM0024069	0.923633
EVM0016224	EVM0009322	0.923633
EVM0025117	EVM0021380	0.923633
EVM0020564	EVM0014706	0.923633
EVM0013047	EVM0006897	0.923633
EVM0008372	EVM0018490	0.923633
EVM0013823	EVM0003916	0.923633
EVM0014706	EVM0000767	0.923633
EVM0011632	EVM0018701	0.923633
EVM0020564	EVM0002082	0.923633
EVM0018643	EVM0012650	0.923633
EVM0009531	EVM0018490	0.923633
EVM0026948	EVM0008210	0.923633
EVM0011820	EVM0000765	0.923633
EVM0026948	EVM0001853	0.923633
EVM0026948	EVM0009554	0.923633
EVM0000541	EVM0003916	0.923633
EVM0022436	EVM0018760	0.923633
EVM0026948	EVM0014706	0.923633
EVM0000541	EVM0024682	0.923633
EVM0023938	EVM0020381	0.923633
EVM0009233	EVM0025117	0.923633
EVM0007391	EVM0028206	0.923633
Colocasia_esculenta_newGene_771	EVM0016194	0.923633
EVM0005581	EVM0007649	0.923633
EVM0012695	EVM0008948	0.923633
EVM0024682	EVM0005180	0.923633
EVM0024682	EVM0001947	0.923633
EVM0012409	EVM0001733	0.923633
EVM0012409	EVM0018411	0.923633
EVM0018490	EVM0019309	0.923633
EVM0018490	EVM0002433	0.923633

EVM0027553	EVM0003916	0.923633
EVM0016010	EVM0015541	0.923633
EVM0010332	EVM0024682	0.923633
EVM0014895	EVM0015270	0.923633
EVM0008210	EVM0010536	0.923633
EVM0001281	EVM0013047	0.923633
EVM0008210	EVM0006304	0.923633
EVM0008210	EVM0012695	0.923633
EVM0014895	EVM0027935	0.923633
EVM0002590	EVM0020628	0.923633
EVM0015270	EVM0015709	0.923633
EVM0002590	EVM0000811	0.923633
EVM0001853	EVM0002433	0.923633
EVM0001853	EVM0009322	0.923633
EVM0002590	EVM0027715	0.923633
EVM0027734	EVM0016728	0.923633
EVM0008210	EVM0013500	0.923633
EVM0026363	EVM0028138	0.923633
Colocasia_esculenta_newGene_462	EVM0002433	0.923633
EVM0010536	EVM0002590	0.923633
EVM0022715	EVM0002082	0.923633
EVM0018760	EVM0001947	0.923633
EVM0010086	EVM0019373	0.923633
EVM0009588	EVM0012360	0.923633
EVM0014597	EVM0004228	0.923633
EVM0016010	EVM0012409	0.923633
Colocasia_esculenta_newGene_863	EVM0027407	0.921569
EVM0023576	EVM0019956	0.921569
EVM0009588	EVM0015709	0.921569
EVM0023918	EVM0023938	0.921569
EVM0020564	EVM0020593	0.921569
EVM0020012	EVM0010905	0.921569
EVM0012607	EVM0010905	0.921569
EVM0010536	EVM0012655	0.921569
EVM0012607	EVM0000767	0.921569
EVM0012607	EVM0008519	0.921569
EVM0003916	EVM0028456	0.921569
EVM0018643	EVM0009322	0.921569
EVM0007391	EVM0020381	0.921569
EVM0026948	EVM0021407	0.921569
EVM0026948	EVM0009588	0.921569
EVM0026948	EVM0010530	0.921569
EVM0026948	EVM0005432	0.921569
EVM0016224	Colocasia_escule	0.921569
EVM0002433	EVM0009322	0.921569
EVM0020628	EVM0013500	0.921569
EVM0003656	EVM0006551	0.921569
EVM0010086	EVM0010905	0.921569
Colocasia_esculenta_newGene_901	EVM0022436	0.921569
EVM0010332	EVM0001853	0.921569
EVM0011820	EVM0010982	0.921569
EVM0026948	EVM0026358	0.921569
EVM0005345	EVM0014895	0.921569
EVM0024474	EVM0020564	0.921569
Colocasia_esculenta_newGene_956	EVM0001739	0.921569
EVM0015709	EVM0001096	0.921569
EVM0018411	EVM0023970	0.921569
EVM0016224	EVM0015709	0.921569

EVM0016224	EVM0017550	0.921569
EVM0000811	EVM0013500	0.921569
EVM0003916	EVM0018701	0.921569
EVM0007214	EVM0025117	0.921569
EVM0007214	Colocasia_escule	0.921569
EVM0026177	EVM0023082	0.921569
EVM0015270	EVM0010905	0.921569
EVM0016224	EVM0014369	0.921569
EVM0016606	EVM0027189	0.921569
EVM0018701	EVM0024069	0.921569
EVM0000767	EVM0010982	0.921569
EVM0020012	EVM0012650	0.921569
EVM0013823	Colocasia_escule	0.921569
EVM0019373	EVM0010905	0.921569
Colocasia_esculenta_newGene_956	EVM0002590	0.921569
EVM0012607	EVM0008474	0.921569
EVM0008210	EVM0006551	0.921569
Colocasia_esculenta_newGene_647	EVM0001739	0.921569
EVM0012607	EVM0014895	0.921569
EVM0010086	EVM0023082	0.921569
EVM0010536	EVM0019956	0.921569
EVM0012607	EVM0019373	0.921569
EVM0021407	EVM0012360	0.921569
EVM0014597	EVM0026358	0.921569
EVM0028456	EVM0005180	0.921569
EVM0007391	EVM0011473	0.921569
EVM0007391	EVM0019956	0.921569
EVM0007391	EVM0012655	0.921569
EVM0014597	EVM0016010	0.921569
EVM0007391	EVM0012650	0.921569
EVM0018411	EVM0018701	0.921569
EVM0009554	EVM0012409	0.921569
EVM0007391	EVM0009174	0.921569
EVM0012695	EVM0027935	0.921569
EVM0028456	EVM0007856	0.921569
EVM0024682	EVM0009322	0.921569
EVM0015270	EVM0028456	0.921569
EVM0028456	EVM0018701	0.921569
EVM0007391	Colocasia_escule	0.921569
EVM0020216	EVM0027734	0.921569
EVM0022133	Colocasia_escule	0.921569
EVM0014597	EVM0023059	0.921569
EVM0008474	EVM0023059	0.921569
EVM0008474	EVM0001853	0.921569
EVM0006304	EVM0019373	0.921569
EVM0027449	EVM0008474	0.921569
EVM0008474	Colocasia_escule	0.921569
EVM0014742	EVM0022327	0.921569
EVM0026948	EVM0002433	0.921569
EVM0010332	EVM0023082	0.921569
EVM0022436	EVM0028456	0.921569
Colocasia_esculenta_newGene_783	EVM0017284	0.921569
EVM0023938	EVM0001739	0.921569
EVM0025628	EVM0028138	0.921569
EVM0008210	EVM0005345	0.921569
EVM0008210	EVM0010332	0.921569
EVM0023059	EVM0001739	0.921569
EVM0014369	EVM0012655	0.921569

EVM0005180	EVM0024069	0.921569
EVM0000541	EVM0009322	0.921569
EVM0025628	EVM0009233	0.921569
EVM0000541	EVM0006551	0.921569
EVM0005432	Colocasia_escule	0.921569
EVM0022436	EVM0001947	0.921569
EVM0014597	EVM0010332	0.919505
EVM0014597	EVM0016596	0.919505
EVM0014597	EVM0026177	0.919505
EVM0023059	EVM0013500	0.919505
EVM0007391	EVM0008519	0.919505
EVM0007391	EVM0015709	0.919505
EVM0014895	EVM0009554	0.919505
EVM0007391	EVM0007649	0.919505
EVM0009554	EVM0005018	0.919505
EVM0004594	EVM0012409	0.919505
EVM0016224	EVM0022436	0.919505
EVM0007649	Colocasia_escule	0.919505
EVM0009531	EVM0010905	0.919505
EVM0007391	EVM0007856	0.919505
EVM0014895	EVM0023082	0.919505
EVM0015709	EVM0008519	0.919505
EVM0025628	EVM0012695	0.919505
EVM0025628	EVM0014895	0.919505
EVM0025628	EVM0025117	0.919505
EVM0012409	EVM0016156	0.919505
EVM0009554	EVM0001096	0.919505
EVM0020628	EVM0012655	0.919505
EVM0008474	EVM0020381	0.919505
Colocasia_esculenta_newGene_783	EVM0012650	0.919505
EVM0010086	EVM0012409	0.919505
EVM0001224	EVM0002082	0.919505
EVM0000811	EVM0012655	0.919505
EVM0005345	EVM0009588	0.919505
Colocasia_esculenta_newGene_865	EVM0020628	0.919505
Colocasia_esculenta_newGene_865	EVM0000811	0.919505
EVM0024474	EVM0001853	0.919505
EVM0002433	EVM0023576	0.919505
EVM0019373	Colocasia_escule	0.919505
Colocasia_esculenta_newGene_901	EVM0024682	0.919505
EVM0005345	EVM0012360	0.919505
EVM0027449	EVM0018411	0.919505
EVM0027449	EVM0015270	0.919505
EVM0026358	EVM0014706	0.919505
EVM0015080	Colocasia_escule	0.919505
EVM0022133	EVM0002590	0.919505
EVM0020216	EVM0002590	0.919505
EVM0022715	EVM0007856	0.919505
EVM0022715	EVM0023576	0.919505
EVM0018760	EVM0020748	0.919505
EVM0027715	EVM0013500	0.919505
EVM0001853	EVM0012655	0.919505
EVM0008210	EVM0028456	0.919505
EVM0027734	EVM0024069	0.919505
EVM0008210	EVM0027935	0.919505
EVM0010332	EVM0019373	0.919505
EVM0008210	EVM0023082	0.919505
EVM0008602	EVM0002433	0.919505

EVM0028456	EVM0004228	0.919505
EVM0019229	EVM0016728	0.919505
EVM0028456	EVM0018643	0.919505
EVM0019229	EVM0000541	0.919505
EVM0010332	EVM0002433	0.919505
EVM0008474	EVM0012655	0.919505
EVM0018490	EVM0000811	0.919505
EVM0018490	EVM0022327	0.919505
EVM0018490	EVM0020628	0.919505
EVM0016224	EVM0011473	0.919505
EVM0016224	EVM0007532	0.919505
EVM0025117	EVM0011820	0.919505
EVM0011820	EVM0020012	0.919505
EVM0020564	EVM0006551	0.919505
EVM0012607	EVM0028456	0.919505
EVM0018643	EVM0019956	0.919505
EVM0027935	EVM0008602	0.919505
EVM0012316	EVM0015541	0.919505
EVM0023082	EVM0001739	0.919505
EVM0000541	EVM0004228	0.919505
EVM0026948	EVM0006551	0.919505
EVM0000541	EVM0023576	0.919505
EVM0023082	EVM0002433	0.919505
EVM0005432	EVM0001853	0.919505
EVM0014369	EVM0013669	0.919505
EVM0007090	EVM0019309	0.919505
EVM0005581	EVM0018411	0.919505
EVM0021407	EVM0026358	0.919505
EVM0020593	EVM0019309	0.919505
EVM0020593	EVM0003656	0.919505
EVM0010530	EVM0013669	0.919505
EVM0010530	EVM0011330	0.919505
EVM0009233	EVM0012695	0.919505
EVM0012695	EVM0022327	0.919505
EVM0020012	EVM0002433	0.917441
EVM0016010	EVM0005180	0.917441
EVM0011820	EVM0008948	0.917441
EVM0016194	EVM0010905	0.917441
EVM0011820	Colocasia_escule	0.917441
EVM0002590	EVM0014369	0.917441
EVM0019373	EVM0028138	0.917441
EVM0022133	EVM0015270	0.917441
Colocasia_esculenta_newGene_863	EVM0026358	0.917441
EVM0006304	EVM0005180	0.917441
EVM0006304	EVM0007532	0.917441
EVM0020012	EVM0016156	0.917441
Colocasia_esculenta_newGene_956	EVM0005180	0.917441
EVM0023059	EVM0024682	0.917441
EVM0027715	EVM0015709	0.917441
EVM0010536	EVM0006304	0.917441
EVM0010530	EVM0020564	0.917441
Colocasia_esculenta_newGene_771	EVM0020593	0.917441
EVM0023082	EVM0012409	0.917441
Colocasia_esculenta_newGene_863	EVM0001733	0.917441
EVM0011820	EVM0005844	0.917441
EVM0023082	EVM0000541	0.917441
EVM0006304	EVM0005432	0.917441
EVM0016010	EVM0008948	0.917441

EVM0016010	EVM0004228	0.917441
EVM0027553	EVM0020308	0.917441
Colocasia_esculenta_newGene_239	EVM0010905	0.917441
EVM0005432	EVM0023970	0.917441
EVM0016010	EVM0021848	0.917441
EVM0012695	Colocasia_escule	0.917441
EVM0016010	EVM0005844	0.917441
EVM0020012	EVM0018411	0.917441
EVM0016194	EVM0008519	0.917441
EVM0016194	EVM0012360	0.917441
EVM0016563	EVM0027734	0.917441
EVM0000541	EVM0002082	0.917441
EVM0007214	EVM0028138	0.917441
EVM0007214	EVM0008948	0.917441
EVM0016224	EVM0000767	0.917441
Colocasia_esculenta_newGene_901	EVM0028456	0.917441
EVM0016224	EVM0008570	0.917441
EVM0016224	EVM0000541	0.917441
EVM0016224	EVM0006304	0.917441
Colocasia_esculenta_newGene_901	EVM0007856	0.917441
EVM0016224	EVM0020216	0.917441
EVM0007391	EVM0007532	0.917441
EVM0012316	EVM0003916	0.917441
EVM0012316	EVM0002433	0.917441
EVM0027935	EVM0005180	0.917441
EVM0020564	EVM0013500	0.917441
EVM0007391	EVM0003656	0.917441
EVM0020381	EVM0001739	0.917441
EVM0009322	EVM0001739	0.917441
EVM0010086	EVM0009174	0.917441
EVM0003916	EVM0020748	0.917441
EVM0020593	EVM0018643	0.917441
EVM0005345	EVM0013823	0.917441
EVM0014369	EVM0007532	0.917441
EVM0010332	EVM0010982	0.917441
EVM0010332	EVM0009322	0.917441
EVM0026948	EVM0022133	0.917441
EVM0012607	EVM0020593	0.917441
EVM0012607	EVM0005018	0.917441
EVM0010332	EVM0004228	0.917441
EVM0021407	EVM0009531	0.917441
EVM0023576	EVM0020748	0.917441
EVM0012607	EVM0027715	0.917441
EVM0012607	EVM0016010	0.917441
EVM0007090	EVM0022327	0.917441
EVM0012607	EVM0022436	0.917441
EVM0010332	EVM0023403	0.917441
EVM0026115	EVM0020308	0.917441
EVM0026115	EVM0001096	0.917441
EVM0015080	Colocasia_escule	0.917441
EVM0010332	EVM0012409	0.917441
EVM0012607	EVM0012316	0.917441
EVM0022715	EVM0012655	0.917441
EVM0011632	EVM0011473	0.917441
EVM0008474	EVM0016606	0.917441
EVM0006551	EVM0010982	0.917441
EVM0009531	EVM0016194	0.917441
EVM0008474	EVM0000541	0.917441

EVM0008474	EVM0013669	0.917441
EVM0007856	EVM0002082	0.917441
EVM0023918	EVM0013713	0.917441
EVM0007649	EVM0018411	0.917441
EVM0007649	EVM0013713	0.917441
EVM0025803	EVM0021380	0.917441
EVM0027734	EVM0001739	0.917441
EVM0008210	EVM0018643	0.917441
EVM0014895	EVM0023403	0.917441
EVM0026363	EVM0008210	0.917441
EVM0013500	EVM0028206	0.917441
EVM0014895	EVM0008519	0.917441
EVM0014895	EVM0016156	0.917441
EVM0016008	EVM0020748	0.917441
EVM0007391	EVM0022436	0.917441
EVM0020564	EVM0013713	0.917441
Colocasia_esculenta_newGene_647	Colocasia_escule	0.917441
EVM0000765	EVM0012650	0.917441
EVM0013823	EVM0027407	0.917441
EVM0025117	EVM0012695	0.917441
EVM0000767	EVM0020748	0.917441
Colocasia_esculenta_newGene_647	EVM0022436	0.917441
EVM0013823	EVM0010982	0.917441
EVM0025117	EVM0014895	0.917441
EVM0013823	Colocasia_escule	0.917441
EVM0015080	EVM0008210	0.915377
EVM0020216	EVM0018411	0.915377
EVM0008570	EVM0019956	0.915377
EVM0012695	EVM0004594	0.915377
EVM0010086	EVM0014895	0.915377
EVM0008210	EVM0020748	0.915377
EVM0005581	EVM0001739	0.915377
EVM0024682	EVM0020748	0.915377
EVM0005581	EVM0020748	0.915377
EVM0012695	EVM0003916	0.915377
EVM0005581	EVM0020381	0.915377
EVM0010086	EVM0007649	0.915377
EVM0008210	EVM0008602	0.915377
EVM0008210	EVM0026358	0.915377
EVM0008210	EVM0027715	0.915377
EVM0008210	EVM0005432	0.915377
EVM0028456	EVM0012650	0.915377
Colocasia_esculenta_newGene_865	EVM0015270	0.915377
EVM0019229	EVM0022715	0.915377
Colocasia_esculenta_newGene_783	EVM0020593	0.915377
Colocasia_esculenta_newGene_865	EVM0019373	0.915377
EVM0022436	EVM0011473	0.915377
EVM0027449	EVM0010086	0.915377
EVM0027449	Colocasia_escule	0.915377
EVM0018490	EVM0005180	0.915377
EVM0027553	EVM0012650	0.915377
EVM0007391	EVM0023970	0.915377
EVM0007391	EVM0023576	0.915377
Colocasia_esculenta_newGene_863	EVM0020748	0.915377
EVM0007391	EVM0027734	0.915377
EVM0000767	EVM0006551	0.915377
EVM0014597	EVM0008519	0.915377
EVM0014597	EVM0024069	0.915377

EVM0009174	EVM0012650	0.915377
EVM0020748	EVM0002082	0.915377
EVM0014597	EVM0016563	0.915377
Colocasia_esculenta_newGene_863	EVM0002524	0.915377
EVM0015270	Colocasia_escule	0.915377
EVM0012650	EVM0005180	0.915377
EVM0001281	EVM0010536	0.915377
EVM0015270	EVM0021848	0.915377
EVM0015270	EVM0002082	0.915377
Colocasia_esculenta_newGene_863	EVM0018411	0.915377
EVM0023918	EVM0015270	0.915377
EVM0002082	EVM0007532	0.915377
EVM0011473	EVM0024069	0.915377
EVM0026363	Colocasia_escule	0.915377
EVM0026363	EVM0020628	0.915377
EVM0026363	EVM0015709	0.915377
EVM0026363	EVM0000811	0.915377
EVM0022436	EVM0000767	0.915377
EVM0012695	EVM0000767	0.915377
EVM0005581	EVM0008474	0.915377
EVM0005581	EVM0020216	0.915377
EVM0026948	EVM0011473	0.915377
EVM0026948	EVM0000767	0.915377
EVM0015541	EVM0002524	0.915377
EVM0026948	EVM0004228	0.915377
EVM0005432	EVM0008570	0.915377
EVM0012607	EVM0024069	0.915377
EVM0012607	EVM0012655	0.915377
Colocasia_esculenta_newGene_570	EVM0017284	0.915377
EVM0012607	EVM0001096	0.915377
EVM0023576	EVM0009322	0.915377
EVM0023576	EVM0007532	0.915377
EVM0020012	EVM0018643	0.915377
EVM0012607	EVM0009531	0.915377
EVM0014706	EVM0027189	0.915377
EVM0026177	EVM0018701	0.915377
EVM0026177	EVM0027407	0.915377
EVM0016224	EVM0014706	0.915377
EVM0016224	EVM0027449	0.915377
EVM0013713	EVM0002082	0.915377
EVM0007391	EVM0009322	0.915377
EVM0010332	EVM0001739	0.915377
EVM0011820	EVM0021380	0.915377
EVM0018411	EVM0012655	0.915377
EVM0006304	EVM0001853	0.915377
EVM0020381	EVM0007532	0.915377
EVM0020564	EVM0012655	0.915377
EVM0018411	EVM0010982	0.915377
EVM0011820	EVM0016010	0.915377
EVM0013823	EVM0005844	0.915377
EVM0016606	EVM0012650	0.915377
EVM0010536	EVM0007532	0.915377
EVM0010332	EVM0014895	0.915377
EVM0003916	EVM0024682	0.915377
EVM0010536	EVM0024069	0.915377
EVM0011632	EVM0013713	0.915377
EVM0003916	EVM0015541	0.915377
Colocasia_esculenta_newGene_647	EVM0002433	0.915377

EVM0021407	Colocasia_escule	0.915377
EVM0021407	EVM0008372	0.915377
Colocasia_esculenta_newGene_771	EVM0008519	0.915377
EVM0018411	EVM0008602	0.915377
EVM0021407	EVM0015709	0.915377
EVM0005345	EVM0024682	0.915377
EVM0023082	EVM0026358	0.915377
EVM0022133	EVM0004228	0.915377
EVM0021407	EVM0000767	0.915377
EVM0005345	EVM0022436	0.915377
EVM0000541	EVM0020628	0.915377
EVM0010530	EVM0018411	0.915377
EVM0000541	EVM0000811	0.915377
EVM0001224	EVM0000541	0.915377
EVM0023082	EVM0022327	0.915377
EVM0011820	EVM0012650	0.915377
EVM0020381	EVM0012650	0.915377
EVM0016596	EVM0007090	0.915377
EVM0022715	EVM0004228	0.915377
EVM0022715	EVM0028456	0.915377
EVM0010536	EVM0014369	0.915377
EVM0022715	EVM0020748	0.915377
EVM0002590	Colocasia_escule	0.915377
EVM0027715	EVM0024682	0.915377
EVM0027715	EVM0020308	0.915377
Colocasia_esculenta_newGene_956	EVM0024069	0.915377
EVM0025980	EVM0001733	0.915377
EVM0014895	EVM0019373	0.915377
EVM0012409	EVM0000541	0.915377
EVM0009531	EVM0009554	0.915377
EVM0027715	EVM0000541	0.915377
EVM0022715	EVM0027189	0.915377
EVM0022715	EVM0002590	0.915377
EVM0009554	EVM0024682	0.915377
Colocasia_esculenta_newGene_956	EVM0023059	0.915377
EVM0016224	EVM0019956	0.913313
EVM0007483	EVM0000767	0.913313
EVM0016224	EVM0017284	0.913313
EVM0007214	EVM0012409	0.913313
EVM0016224	EVM0023970	0.913313
EVM0025980	EVM0012409	0.913313
EVM0000541	Colocasia_escule	0.913313
EVM0016224	EVM0004228	0.913313
EVM0000541	EVM0012655	0.913313
Colocasia_esculenta_newGene_462	EVM0007856	0.913313
EVM0014895	EVM0002082	0.913313
EVM0021407	EVM0016010	0.913313
Colocasia_esculenta_newGene_771	EVM0015709	0.913313
EVM0007090	EVM0018490	0.913313
EVM0024069	EVM0001739	0.913313
EVM0014706	EVM0015709	0.913313
EVM0020012	EVM0022327	0.913313
EVM0012607	EVM0020381	0.913313
EVM0010536	EVM0018411	0.913313
EVM0020308	EVM0012650	0.913313
EVM0020308	EVM0010982	0.913313
EVM0010530	EVM0024069	0.913313
EVM0010530	EVM0017284	0.913313

EVM0007214	EVM0010982	0.913313
EVM0007214	EVM0012650	0.913313
EVM0025980	EVM0010982	0.913313
EVM0011632	EVM0024351	0.913313
EVM0015270	EVM0000811	0.913313
EVM0010536	EVM0005180	0.913313
Colocasia_esculenta_newGene_863	EVM0022327	0.913313
EVM0014597	EVM0012695	0.913313
Colocasia_esculenta_newGene_863	EVM0021380	0.913313
EVM0011632	EVM0014706	0.913313
EVM0014597	EVM0008570	0.913313
EVM0001281	EVM0006551	0.913313
EVM0014597	EVM0002082	0.913313
EVM0016606	EVM0007532	0.913313
Colocasia_esculenta_newGene_863	EVM0000767	0.913313
EVM0015270	Colocasia_escule	0.913313
EVM0016606	EVM0024682	0.913313
EVM0011820	EVM0012695	0.913313
EVM0007391	EVM0023059	0.913313
EVM0013823	EVM0012409	0.913313
EVM0007391	EVM0000541	0.913313
EVM0015270	EVM0020628	0.913313
EVM0020564	EVM0007856	0.913313
EVM0009322	EVM0002082	0.913313
EVM0023918	EVM0001739	0.913313
EVM0016596	EVM0010905	0.913313
EVM0020564	EVM0011473	0.913313
EVM0007391	EVM0006551	0.913313
EVM0000541	EVM0028456	0.913313
EVM0007649	EVM0001739	0.913313
EVM0025628	Colocasia_escule	0.913313
EVM0023918	EVM0002433	0.913313
EVM0023082	EVM0015709	0.913313
EVM0000541	EVM0016728	0.913313
EVM0023938	EVM0018643	0.913313
Colocasia_esculenta_newGene_865	EVM0018411	0.913313
EVM0006304	EVM0027189	0.913313
EVM0019373	EVM0018411	0.913313
EVM0002433	EVM0015709	0.913313
Colocasia_esculenta_newGene_901	EVM0000767	0.913313
Colocasia_esculenta_newGene_901	EVM0009322	0.913313
EVM0008602	EVM0018643	0.913313
EVM0010332	EVM0023059	0.913313
EVM0010332	EVM0009554	0.913313
EVM0015080	EVM0000765	0.913313
EVM0008570	EVM0012655	0.913313
EVM0020216	EVM0020628	0.913313
EVM0022436	EVM0018701	0.913313
EVM0020216	EVM0000811	0.913313
EVM0010332	EVM0014369	0.913313
EVM0024682	EVM0014369	0.913313
EVM0006304	EVM0023059	0.913313
EVM0005581	EVM0009174	0.913313
EVM0024682	EVM0007856	0.913313
EVM0022133	EVM0024069	0.913313
EVM0008210	EVM0007856	0.913313
EVM0027734	Colocasia_escule	0.913313
EVM0008210	EVM0013713	0.913313

EVM0008210	EVM0020628	0.913313
EVM0008210	EVM0000811	0.913313
EVM0001853	EVM0001739	0.913313
EVM0002590	EVM0014706	0.913313
EVM0025803	EVM0015541	0.913313
EVM0022715	EVM0005432	0.913313
EVM0019229	EVM0012650	0.913313
EVM0002590	EVM0008519	0.913313
EVM0027553	EVM0016010	0.913313
EVM0008474	EVM0008519	0.913313
EVM0028456	EVM0008519	0.913313
EVM0008474	Colocasia_escule	0.913313
EVM0022715	EVM0005180	0.913313
Colocasia_esculenta_newGene_647	EVM0002590	0.913313
EVM0024474	EVM0014369	0.913313
Colocasia_esculenta_newGene_865	EVM0015709	0.913313
Colocasia_esculenta_newGene_865	EVM0018490	0.913313
EVM0018643	EVM0023576	0.913313
EVM0016563	EVM0001739	0.913313
EVM0005345	EVM0011330	0.913313
EVM0015541	EVM0028206	0.913313
EVM0018411	EVM0028456	0.913313
Colocasia_esculenta_newGene_570	EVM0000767	0.913313
EVM0005432	EVM0024069	0.913313
EVM0026948	EVM0023576	0.913313
EVM0005432	EVM0018760	0.913313
EVM0022436	EVM0002433	0.913313
EVM0022133	EVM0003656	0.913313
EVM0026948	EVM0005180	0.913313
EVM0005345	Colocasia_escule	0.913313
EVM0022436	EVM0007483	0.913313
EVM0026948	EVM0001739	0.913313
EVM0021407	Colocasia_escule	0.913313
EVM0022436	EVM0012650	0.913313
EVM0005581	EVM0015270	0.913313
EVM0005581	EVM0022133	0.913313
EVM0005581	EVM0019229	0.913313
EVM0018411	EVM0017284	0.913313
EVM0001224	EVM0006551	0.913313
EVM0026358	EVM0024682	0.913313
EVM0016563	EVM0024682	0.913313
EVM0022133	EVM0000767	0.913313
EVM0009233	Colocasia_escule	0.913313
EVM0009233	EVM0021407	0.913313
EVM0027734	EVM0028456	0.911249
Colocasia_esculenta_newGene_570	EVM0012650	0.911249
EVM0001853	EVM0003656	0.911249
EVM0027734	EVM0012650	0.911249
Colocasia_esculenta_newGene_863	EVM0026177	0.911249
EVM0000541	EVM0008519	0.911249
Colocasia_esculenta_newGene_863	EVM0016563	0.911249
EVM0019956	EVM0007532	0.911249
Colocasia_esculenta_newGene_570	EVM0028206	0.911249
EVM0026358	EVM0010982	0.911249
EVM0016563	EVM0019956	0.911249
EVM0009554	EVM0010905	0.911249
EVM0018411	EVM0000767	0.911249
EVM0018411	EVM0002082	0.911249

EVM0022327	EVM0016156	0.911249
EVM0000765	EVM0020748	0.911249
EVM0018411	EVM0013500	0.911249
EVM0014369	EVM0023970	0.911249
EVM0016563	EVM0000541	0.911249
EVM0015709	EVM0020628	0.911249
EVM0016563	EVM0012650	0.911249
EVM0000811	EVM0015709	0.911249
Colocasia_esculenta_newGene_783	EVM0018643	0.911249
EVM0018411	EVM0016728	0.911249
EVM0003916	EVM0008519	0.911249
EVM0015541	EVM0012650	0.911249
EVM0018490	EVM0015709	0.911249
EVM0012409	Colocasia_escule	0.911249
EVM0000541	EVM0010982	0.911249
EVM0007856	EVM0013500	0.911249
EVM0007856	EVM0007532	0.911249
EVM0023938	EVM0000541	0.911249
EVM0016224	EVM0013047	0.911249
EVM0025980	Colocasia_escule	0.911249
EVM0016224	EVM0005581	0.911249
EVM0019229	EVM0006551	0.911249
EVM0014895	EVM0016010	0.911249
EVM0007391	EVM0008210	0.911249
EVM0021407	EVM0021848	0.911249
EVM0014895	EVM0005844	0.911249
EVM0010536	EVM0013713	0.911249
EVM0014597	EVM0012316	0.911249
EVM0007391	EVM0019373	0.911249
EVM0013047	EVM0020564	0.911249
EVM0007391	EVM0020012	0.911249
EVM0012607	EVM0020564	0.911249
EVM0008210	EVM0018490	0.911249
EVM0007391	EVM0023082	0.911249
EVM0020012	EVM0010982	0.911249
EVM0008210	EVM0008948	0.911249
EVM0027553	EVM0016606	0.911249
EVM0009531	Colocasia_escule	0.911249
EVM0009531	EVM0001096	0.911249
EVM0011632	EVM0018411	0.911249
EVM0025117	EVM0016010	0.911249
EVM0013823	EVM0007483	0.911249
EVM0016596	EVM0018490	0.911249
EVM0020381	EVM0013500	0.911249
EVM0001281	EVM0022715	0.911249
EVM0020381	EVM0019373	0.911249
EVM0007649	EVM0002433	0.911249
EVM0019373	EVM0026358	0.911249
EVM0026948	EVM0007856	0.911249
EVM0010086	EVM0010332	0.911249
Colocasia_esculenta_newGene_865	EVM0014369	0.911249
EVM0014597	EVM0019309	0.911249
EVM0014597	EVM0005844	0.911249
EVM0012316	EVM0023059	0.911249
EVM0025980	EVM0012695	0.911249
EVM0008474	EVM0007532	0.911249
EVM0022715	EVM0010530	0.911249
EVM0006304	EVM0014369	0.911249

EVM0022715	EVM0017550	0.911249
EVM0015080	EVM0010982	0.911249
EVM0011820	EVM0020308	0.911249
EVM0005345	EVM0015270	0.911249
Colocasia_esculenta_newGene_239	EVM0002433	0.911249
EVM0010332	EVM0010530	0.911249
EVM0011820	EVM0023403	0.911249
EVM0004594	EVM0000765	0.911249
EVM0022715	EVM0012650	0.911249
Colocasia_esculenta_newGene_771	EVM0005018	0.911249
EVM0010332	Colocasia_escule	0.911249
EVM0013047	EVM0024351	0.911249
EVM0006304	EVM0004228	0.911249
EVM0023918	EVM0010086	0.911249
EVM0023059	EVM0007532	0.911249
EVM0022133	EVM0024682	0.911249
EVM0008210	EVM0024069	0.911249
EVM0027449	EVM0022133	0.911249
EVM0010332	EVM0018760	0.911249
EVM0012607	EVM0025117	0.911249
EVM0012607	EVM0020012	0.911249
EVM0023059	EVM0018643	0.911249
Colocasia_esculenta_newGene_647	Colocasia_escule	0.911249
EVM0005345	EVM0008519	0.911249
Colocasia_esculenta_newGene_956	EVM0008570	0.911249
EVM0020628	EVM0006551	0.909185
EVM0010086	EVM0015541	0.909185
EVM0000811	EVM0006551	0.909185
EVM0016596	EVM0002524	0.909185
EVM0012409	EVM0008519	0.909185
EVM0010530	EVM0014706	0.909185
EVM0014597	EVM0020564	0.909185
EVM0022133	EVM0022436	0.909185
EVM0014597	EVM0027935	0.909185
EVM0021390	Colocasia_escule	0.909185
EVM0009531	EVM0008519	0.909185
EVM0008570	EVM0009322	0.909185
EVM0022133	EVM0018411	0.909185
EVM0013047	EVM0025803	0.909185
EVM0027449	EVM0009174	0.909185
EVM0016606	EVM0018643	0.909185
EVM0027449	EVM0003656	0.909185
Colocasia_esculenta_newGene_901	EVM0010332	0.909185
EVM0010536	EVM0017284	0.909185
Colocasia_esculenta_newGene_901	EVM0000765	0.909185
EVM0014895	EVM0000767	0.909185
EVM0009554	EVM0010982	0.909185
EVM0014895	EVM0010905	0.909185
EVM0012409	EVM0027935	0.909185
EVM0002433	EVM0008519	0.909185
EVM0004228	EVM0024682	0.909185
EVM0012409	EVM0023403	0.909185
EVM0010530	EVM0024474	0.909185
EVM0009588	EVM0018760	0.909185
EVM0004228	EVM0010982	0.909185
EVM0010530	EVM0000541	0.909185
EVM0005018	EVM0026115	0.909185
EVM0027734	EVM0007856	0.909185

EVM0010905	EVM0019309	0.909185
EVM0017284	EVM0013500	0.909185
EVM0027734	EVM0013713	0.909185
EVM0026358	EVM0028138	0.909185
EVM0005345	EVM0007483	0.909185
EVM0027734	EVM0008570	0.909185
EVM0018411	EVM0027734	0.909185
EVM0018411	EVM0024682	0.909185
EVM0018411	EVM0020593	0.909185
EVM0018411	EVM0014369	0.909185
EVM0014578	EVM0005180	0.909185
EVM0018760	EVM0018701	0.909185
EVM0018760	EVM0006551	0.909185
EVM0001224	EVM0022436	0.909185
EVM0022715	EVM0017284	0.909185
EVM0022715	EVM0011473	0.909185
Colocasia_esculenta_newGene_647	EVM0010332	0.909185
EVM0010332	EVM0005432	0.909185
EVM0007391	EVM0004228	0.909185
EVM0007391	EVM0008570	0.909185
EVM0001947	EVM0024069	0.909185
EVM0007391	EVM0013669	0.909185
EVM0003916	EVM0011330	0.909185
Colocasia_esculenta_newGene_647	EVM0023082	0.909185
Colocasia_esculenta_newGene_647	EVM0007532	0.909185
EVM0020564	EVM0014369	0.909185
EVM0011632	EVM0006551	0.909185
EVM0016008	Colocasia_escule	0.909185
EVM0016008	EVM0013713	0.909185
EVM0014597	EVM0028456	0.909185
EVM0013823	EVM0011820	0.909185
EVM0020381	EVM0002082	0.909185
EVM0008474	EVM0022133	0.909185
EVM0013713	EVM0017284	0.909185
EVM0013713	EVM0000767	0.909185
EVM0012316	EVM0000765	0.909185
EVM0027935	EVM0019309	0.909185
EVM0012316	EVM0016728	0.909185
EVM0027935	EVM0001739	0.909185
EVM0012316	Colocasia_escule	0.909185
EVM0012316	EVM0011820	0.909185
EVM0023938	EVM0015270	0.909185
EVM0028456	EVM0013713	0.909185
EVM0025628	EVM0015080	0.909185
EVM0000541	EVM0005180	0.909185
EVM0021407	EVM0002524	0.909185
EVM0025803	EVM0006897	0.909185
EVM0000541	EVM0001739	0.909185
EVM0008210	EVM0022436	0.909185
EVM0025628	EVM0012607	0.909185
EVM0008210	EVM0027734	0.909185
EVM0021407	EVM0016194	0.909185
EVM0012650	EVM0007532	0.909185
EVM0015270	EVM0018490	0.909185
EVM0001281	EVM0023576	0.909185
EVM0002590	EVM0016194	0.909185
EVM0024069	EVM0007532	0.909185
EVM0012655	EVM0005180	0.909185

EVM0005581	EVM0007856	0.909185
EVM0001853	EVM0024682	0.909185
EVM0023059	EVM0012650	0.909185
EVM0023918	EVM0008474	0.909185
EVM0005180	EVM0018701	0.909185
EVM0023918	EVM0010536	0.909185
EVM0001853	EVM0000541	0.909185
EVM0022436	EVM0016606	0.909185
EVM0002590	EVM0023970	0.909185
EVM0021380	EVM0028138	0.909185
EVM0005432	EVM0023082	0.909185
EVM0022436	Colocasia_escule	0.909185
EVM0018643	EVM0002082	0.909185
EVM0012607	EVM0001733	0.909185
EVM0012695	EVM0006897	0.909185
EVM0020593	EVM0002082	0.909185
EVM0023970	EVM0002082	0.909185
EVM0023970	EVM0013713	0.909185
EVM0009233	EVM0012409	0.909185
EVM0014369	EVM0018643	0.909185
EVM0024474	EVM0017550	0.909185
EVM0024474	EVM0014706	0.909185
EVM0019373	EVM0023059	0.909185
EVM0014369	EVM0001739	0.909185
EVM0014369	EVM0015709	0.909185
EVM0012607	EVM0025980	0.909185
Colocasia_esculenta_newGene_462	EVM0010982	0.909185
EVM0012607	EVM0010332	0.909185
EVM0022133	EVM0017550	0.909185
EVM0013669	EVM0007532	0.909185
EVM0020216	EVM0020564	0.909185
Colocasia_esculenta_newGene_956	EVM0001853	0.909185
EVM0016728	EVM0013713	0.909185
EVM0012607	EVM0015080	0.909185
Colocasia_esculenta_newGene_863	EVM0024682	0.909185
EVM0012607	EVM0016596	0.909185
EVM0020216	EVM0022133	0.909185
Colocasia_esculenta_newGene_863	EVM0000541	0.909185
EVM0013669	EVM0019956	0.909185
EVM0006304	EVM0019309	0.909185
Colocasia_esculenta_newGene_956	EVM0020564	0.909185
EVM0000541	EVM0020593	0.907121
EVM0023938	EVM0014369	0.907121
EVM0012695	EVM0001733	0.907121
EVM0007391	EVM0024682	0.907121
EVM0019229	EVM0018411	0.907121
EVM0016224	EVM0012607	0.907121
EVM0019229	EVM0013500	0.907121
EVM0005432	EVM0016563	0.907121
Colocasia_esculenta_newGene_462	EVM0012409	0.907121
EVM0005432	EVM0026358	0.907121
EVM0005432	EVM0000811	0.907121
Colocasia_esculenta_newGene_647	EVM0009322	0.907121
EVM0014369	EVM0001947	0.907121
EVM0008474	EVM0001947	0.907121
EVM0012316	EVM0018643	0.907121
EVM0003916	EVM0021848	0.907121
EVM0003916	EVM0013713	0.907121

EVM0026948	EVM0013713	0.907121
EVM0023970	EVM0001947	0.907121
EVM0026948	EVM0016563	0.907121
EVM0027407	EVM0005844	0.907121
EVM0012316	EVM0018411	0.907121
Colocasia_esculenta_newGene_462	EVM0006551	0.907121
EVM0012316	EVM0023082	0.907121
EVM0026948	Colocasia_escule	0.907121
EVM0026948	EVM0010536	0.907121
EVM0012607	Colocasia_escule	0.907121
EVM0000767	EVM0024069	0.907121
EVM0018411	EVM0000811	0.907121
EVM0010332	EVM0020593	0.907121
EVM0005345	EVM0018411	0.907121
EVM0014578	Colocasia_escule	0.907121
EVM0023918	EVM0016224	0.907121
EVM0026363	EVM0012409	0.907121
Colocasia_esculenta_newGene_239	EVM0015270	0.907121
EVM0018411	EVM0018643	0.907121
EVM0005345	Colocasia_escule	0.907121
EVM0023576	EVM0013500	0.907121
EVM0010332	EVM0018643	0.907121
EVM0018411	EVM0020628	0.907121
Colocasia_esculenta_newGene_863	EVM0008570	0.907121
EVM0015270	EVM0020564	0.907121
EVM0018411	EVM0000765	0.907121
EVM0010332	EVM0007532	0.907121
EVM0026363	EVM0005345	0.907121
EVM0010332	EVM0013500	0.907121
EVM0012607	EVM0009233	0.907121
EVM0005432	EVM0002433	0.907121
Colocasia_esculenta_newGene_863	EVM0027935	0.907121
EVM0005432	EVM0020628	0.907121
EVM0027734	EVM0011473	0.907121
EVM0010332	EVM0024474	0.907121
EVM0005018	EVM0001096	0.907121
EVM0012607	EVM0004228	0.907121
EVM0007214	EVM0001733	0.907121
EVM0010905	EVM0010982	0.907121
EVM0015270	EVM0016156	0.907121
EVM0012607	EVM0016563	0.907121
EVM0014742	EVM0014895	0.907121
EVM0012607	EVM0026177	0.907121
EVM0008210	EVM0000767	0.907121
EVM0007214	Colocasia_escule	0.907121
EVM0003916	EVM0024069	0.907121
EVM0015270	EVM0011473	0.907121
Colocasia_esculenta_newGene_863	Colocasia_escule	0.907121
EVM0023059	EVM0007856	0.907121
EVM0027553	EVM0015270	0.907121
EVM0014597	EVM0016224	0.907121
Colocasia_esculenta_newGene_956	EVM0022436	0.907121
EVM0010086	EVM0006551	0.907121
EVM0005581	EVM0020564	0.907121
EVM0016596	Colocasia_escule	0.907121
Colocasia_esculenta_newGene_783	EVM0009174	0.907121
EVM0016010	EVM0012650	0.907121
EVM0020628	EVM0008519	0.907121

EVM0027715	EVM0007856	0.907121
EVM0027715	EVM0012655	0.907121
Colocasia_esculenta_newGene_956	EVM0000541	0.907121
Colocasia_esculenta_newGene_901	EVM0022715	0.907121
EVM0020216	EVM0007649	0.907121
Colocasia_esculenta_newGene_956	EVM0017284	0.907121
EVM0007649	EVM0008519	0.907121
EVM0009588	EVM0024474	0.907121
EVM0010086	Colocasia_escule	0.907121
EVM0000811	EVM0008519	0.907121
EVM0005581	EVM0018643	0.907121
EVM0002590	Colocasia_escule	0.907121
EVM0016224	EVM0007856	0.907121
Colocasia_esculenta_newGene_901	EVM0009174	0.907121
EVM0012409	EVM0026358	0.907121
EVM0001853	EVM0014369	0.907121
EVM0010536	Colocasia_escule	0.907121
EVM0014895	EVM0018411	0.907121
EVM0014597	Colocasia_escule	0.907121
Colocasia_esculenta_newGene_901	EVM0002082	0.907121
EVM0024682	EVM0007483	0.907121
EVM0009233	EVM0021848	0.907121
EVM0025117	EVM0016596	0.907121
EVM0018490	EVM0012655	0.907121
EVM0027553	EVM0010982	0.907121
EVM0020216	EVM0023970	0.907121
EVM0008474	EVM0023938	0.907121
EVM0008570	EVM0024682	0.907121
EVM0020381	Colocasia_escule	0.907121
EVM0008570	EVM0020593	0.907121
EVM0008474	EVM0007649	0.907121
EVM0014597	EVM0015541	0.907121
EVM0012650	EVM0011473	0.907121
EVM0020381	EVM0028456	0.907121
EVM0023082	EVM0009554	0.907121
EVM0009233	EVM0010086	0.907121
EVM0018490	EVM0004228	0.907121
EVM0008474	EVM0008570	0.907121
EVM0007214	EVM0012316	0.907121
EVM0005581	EVM0010332	0.907121
EVM0027553	EVM0013713	0.907121
EVM0016008	EVM0018411	0.907121
EVM0020216	EVM0015270	0.907121
EVM0022133	EVM0010982	0.907121
EVM0005581	EVM0010086	0.907121
EVM0001281	EVM0006897	0.907121
EVM0015080	EVM0021407	0.905057
Colocasia_esculenta_newGene_647	EVM0019373	0.905057
EVM0015709	EVM0006551	0.905057
EVM0015080	EVM0019373	0.905057
EVM0002433	EVM0028206	0.905057
EVM0008570	EVM0001739	0.905057
EVM0003656	EVM0013713	0.905057
EVM0016224	EVM0010530	0.905057
EVM0002433	EVM0020748	0.905057
EVM0012607	Colocasia_escule	0.905057
EVM0016728	EVM0020748	0.905057
EVM0016224	EVM0002590	0.905057

EVM0027449	EVM0020564	0.905057
EVM0027449	EVM0027734	0.905057
EVM0013669	EVM0007856	0.905057
EVM0022327	EVM0010982	0.905057
EVM0016224	EVM0027734	0.905057
EVM0001947	EVM0002082	0.905057
EVM0001947	EVM0018701	0.905057
EVM0002524	EVM0028138	0.905057
EVM0012607	EVM0028138	0.905057
EVM0008210	Colocasia_escule	0.905057
EVM0012607	EVM0020748	0.905057
Colocasia_esculenta_newGene_783	EVM0013713	0.905057
EVM0005581	Colocasia_escule	0.905057
EVM0012607	EVM0018701	0.905057
EVM0020593	EVM0019956	0.905057
EVM0010086	EVM0010982	0.905057
EVM0028456	EVM0017284	0.905057
EVM0016008	EVM0027935	0.905057
EVM0019229	EVM0016606	0.905057
EVM0018643	EVM0012655	0.905057
EVM0008474	EVM0019956	0.905057
EVM0009233	EVM0013823	0.905057
EVM0016224	EVM0011632	0.905057
EVM0008474	EVM0009322	0.905057
EVM0008474	EVM0016563	0.905057
EVM0028456	EVM0007532	0.905057
EVM0016224	EVM0020593	0.905057
EVM0005581	EVM0013500	0.905057
EVM0016224	EVM0005180	0.905057
EVM0012607	EVM0012695	0.905057
EVM0008210	EVM0002082	0.905057
EVM0010086	EVM0022133	0.905057
EVM0010086	EVM0012695	0.905057
EVM0005581	EVM0016728	0.905057
EVM0023576	EVM0012650	0.905057
EVM0012607	EVM0009554	0.905057
EVM0016224	EVM0001739	0.905057
EVM0024682	EVM0011473	0.905057
EVM0012607	EVM0008570	0.905057
EVM0012607	EVM0000765	0.905057
EVM0027734	EVM0019956	0.905057
EVM0010086	Colocasia_escule	0.905057
EVM0005581	EVM0027734	0.905057
EVM0023970	EVM0003656	0.905057
EVM0020593	Colocasia_escule	0.905057
Colocasia_esculenta_newGene_783	EVM0001739	0.905057
EVM0001281	EVM0010332	0.905057
EVM0016010	EVM0028456	0.905057
EVM0016596	EVM0028138	0.905057
EVM0016596	EVM0027935	0.905057
EVM0016596	EVM0023082	0.905057
EVM0020564	EVM0008602	0.905057
EVM0007856	EVM0018701	0.905057
EVM0007856	EVM0011473	0.905057
EVM0020564	EVM0001947	0.905057
EVM0025117	EVM0021390	0.905057
EVM0007856	EVM0001739	0.905057
EVM0020381	EVM0016606	0.905057

EVM0019373	EVM0016606	0.905057
EVM0019373	EVM0027935	0.905057
EVM0027935	Colocasia_escule	0.905057
EVM0019373	EVM0020308	0.905057
EVM0019373	EVM0001739	0.905057
EVM0019373	EVM0008519	0.905057
EVM0001281	EVM0019229	0.905057
EVM0001281	EVM0020216	0.905057
EVM0023938	Colocasia_escule	0.905057
EVM0012695	EVM0008570	0.905057
EVM0023918	EVM0018643	0.905057
EVM0022133	EVM0018760	0.905057
EVM0022133	EVM0016606	0.905057
EVM0012650	EVM0001739	0.905057
EVM0011820	EVM0001733	0.905057
Colocasia_esculenta_newGene_956	EVM0028456	0.905057
EVM0001281	EVM0014706	0.905057
EVM0011820	EVM0015541	0.905057
EVM0016606	EVM0023403	0.905057
EVM0016606	EVM0016728	0.905057
EVM0002590	EVM0022436	0.905057
EVM0016606	EVM0020593	0.905057
EVM0001853	EVM0027189	0.905057
EVM0002590	EVM0016563	0.905057
EVM0011330	EVM0010982	0.905057
EVM0010536	EVM0024682	0.905057
EVM0012655	EVM0013500	0.905057
EVM0002590	EVM0020564	0.905057
EVM0001281	EVM0016606	0.905057
EVM0001853	EVM0016563	0.905057
EVM0014597	EVM0022436	0.905057
EVM0012409	Colocasia_escule	0.905057
EVM0027553	EVM0023059	0.905057
EVM0016010	EVM0008570	0.905057
EVM0021407	EVM0018411	0.905057
EVM0005432	EVM0004228	0.905057
EVM0000765	EVM0010982	0.905057
EVM0020012	EVM0003916	0.905057
EVM0022436	EVM0027715	0.905057
EVM0026358	EVM0015709	0.905057
EVM0017284	EVM0001739	0.905057
EVM0020012	EVM0000765	0.905057
EVM0020012	EVM0028138	0.905057
EVM0016194	EVM0023082	0.905057
EVM0017284	EVM0007856	0.905057
EVM0001224	EVM0027734	0.905057
EVM0027189	EVM0013500	0.905057
EVM0023059	EVM0009322	0.905057
EVM0010332	EVM0012650	0.905057
EVM0015270	EVM0020593	0.905057
Colocasia_esculenta_newGene_863	EVM0008948	0.905057
EVM0008602	EVM0012650	0.905057
Colocasia_esculenta_newGene_462	EVM0009174	0.905057
EVM0010332	EVM0005345	0.905057
EVM0021407	EVM0008948	0.905057
EVM0022436	EVM0013713	0.905057
EVM0000765	EVM0028206	0.905057
EVM0021407	EVM0005844	0.905057

EVM0012695	EVM0020748	0.905057
EVM0018643	EVM0005180	0.902993
EVM0015270	EVM0004228	0.902993
EVM0026363	EVM0021407	0.902993
Colocasia_esculenta_newGene_863	EVM0006897	0.902993
EVM0005581	EVM0028206	0.902993
EVM0002590	EVM0023082	0.902993
EVM0015541	Colocasia_escule	0.902993
EVM0024682	EVM0012360	0.902993
EVM0001281	EVM0024682	0.902993
EVM0022436	EVM0015709	0.902993
EVM0005581	EVM0006551	0.902993
EVM0026948	EVM0012409	0.902993
EVM0015270	EVM0012655	0.902993
EVM0026948	Colocasia_escule	0.902993
EVM0027715	EVM0002433	0.902993
EVM0015270	EVM0028206	0.902993
EVM0027715	EVM0014706	0.902993
EVM0020216	EVM0005432	0.902993
Colocasia_esculenta_newGene_462	EVM0008602	0.902993
EVM0013669	EVM0009322	0.902993
EVM0012650	EVM0024069	0.902993
EVM0006304	EVM0022436	0.902993
EVM0022133	EVM0020748	0.902993
Colocasia_esculenta_newGene_462	EVM0012655	0.902993
EVM0012650	EVM0010982	0.902993
EVM0022133	EVM0014706	0.902993
EVM0012607	EVM0013500	0.902993
EVM0005432	EVM0000541	0.902993
EVM0024474	EVM0013669	0.902993
EVM0026948	EVM0010982	0.902993
EVM0027553	EVM0002082	0.902993
Colocasia_esculenta_newGene_611	EVM0013500	0.902993
EVM0005432	EVM0020593	0.902993
EVM0019373	EVM0014369	0.902993
EVM0027553	EVM0007856	0.902993
EVM0027553	EVM0020748	0.902993
EVM0020593	EVM0027189	0.902993
EVM0014369	EVM0007856	0.902993
EVM0012607	EVM0021380	0.902993
EVM0012607	EVM0007856	0.902993
EVM0023918	EVM0019373	0.902993
EVM0020012	Colocasia_escule	0.902993
EVM0020012	EVM0013713	0.902993
EVM0014369	EVM0024069	0.902993
EVM0016010	Colocasia_escule	0.902993
EVM0012607	EVM0002082	0.902993
EVM0016010	EVM0020748	0.902993
EVM0026948	EVM0012655	0.902993
EVM0002590	EVM0006551	0.902993
EVM0024682	EVM0008519	0.902993
EVM0023918	EVM0020012	0.902993
EVM0023576	EVM0007856	0.902993
EVM0001853	EVM0028456	0.902993
EVM0002590	EVM0000541	0.902993
EVM0005581	EVM0017284	0.902993
EVM0015541	EVM0028138	0.902993
EVM0007391	EVM0023938	0.902993

EVM0021407	EVM0016156	0.902993
EVM0021407	Colocasia_escule	0.902993
EVM0019229	EVM0014369	0.902993
EVM0000541	EVM0023970	0.902993
EVM0023938	EVM0023059	0.902993
EVM0028456	EVM0009322	0.902993
EVM0028456	EVM0005844	0.902993
EVM0023938	EVM0012655	0.902993
EVM0005844	EVM0010982	0.902993
EVM0014597	EVM0000767	0.902993
EVM0027407	EVM0012360	0.902993
EVM0008474	EVM0021848	0.902993
EVM0012316	EVM0017284	0.902993
EVM0012316	EVM0005180	0.902993
EVM0008474	EVM0014369	0.902993
EVM0014597	EVM0020308	0.902993
EVM0020381	Colocasia_escule	0.902993
EVM0020381	EVM0027715	0.902993
EVM0020381	EVM0020593	0.902993
EVM0013823	EVM0021390	0.902993
EVM0014597	EVM0005018	0.902993
EVM0010332	EVM0021407	0.902993
EVM0010332	EVM0007649	0.902993
EVM0010332	EVM0010536	0.902993
EVM0003916	EVM0002082	0.902993
EVM0010332	EVM0026358	0.902993
EVM0012360	EVM0005844	0.902993
EVM0018411	EVM0019956	0.902993
EVM0010332	EVM0017550	0.902993
EVM0010332	EVM0007856	0.902993
EVM0017284	EVM0012650	0.902993
EVM0005345	EVM0016596	0.902993
EVM0007391	EVM0016606	0.902993
EVM0008210	EVM0016156	0.902993
EVM0018411	EVM0018760	0.902993
EVM0005345	EVM0001733	0.902993
EVM0026358	EVM0023970	0.902993
EVM0008210	EVM0014369	0.902993
EVM0021407	EVM0025117	0.902993
EVM0010905	EVM0000767	0.902993
EVM0000765	EVM0001733	0.902993
EVM0021407	EVM0015541	0.902993
EVM0008210	EVM0002590	0.902993
EVM0025980	EVM0000767	0.902993
EVM0002433	Colocasia_escule	0.902993
EVM0014895	EVM0020593	0.902993
EVM0002433	EVM0013669	0.902993
EVM0010536	EVM0000541	0.902993
EVM0010536	EVM0018760	0.902993
EVM0016606	EVM0001739	0.902993
EVM0010536	EVM0007856	0.902993
EVM0016606	EVM0007856	0.902993
EVM0016606	EVM0006551	0.902993
EVM0016606	EVM0020748	0.902993
EVM0016606	Colocasia_escule	0.902993
EVM0027449	EVM0010332	0.902993
EVM0006551	EVM0013500	0.902993
EVM0011820	EVM0002082	0.902993

EVM0015080	EVM0015541	0.902993
EVM0015080	EVM0020012	0.902993
EVM0011820	EVM0028206	0.902993
EVM0023082	EVM0015541	0.902993
EVM0010530	EVM0024682	0.902993
EVM0022133	EVM0000541	0.902993
EVM0008570	EVM0017284	0.902993
EVM0020564	EVM0017550	0.902993
EVM0011632	EVM0020564	0.902993
EVM0011632	EVM0018760	0.902993
Colocasia_esculenta_newGene_783	Colocasia_escule	0.902993
EVM0021390	EVM0027407	0.902993
EVM0021390	EVM0003916	0.902993
EVM0002524	EVM0000765	0.902993
EVM0012409	EVM0002082	0.902993
EVM0010086	Colocasia_escule	0.902993
EVM0012409	EVM0012650	0.902993
EVM0009588	EVM0023082	0.902993
EVM0025980	EVM0011820	0.902993
Colocasia_esculenta_newGene_865	EVM0018643	0.902993
EVM0009588	EVM0024682	0.902993
EVM0009588	EVM0026358	0.902993
EVM0014597	EVM0001733	0.900929
EVM0007391	EVM0014369	0.900929
EVM0026363	EVM0012607	0.900929
EVM0013669	EVM0011473	0.900929
EVM0016224	EVM0023082	0.900929
EVM0026363	EVM0015080	0.900929
EVM0012650	EVM0002082	0.900929
EVM0007391	EVM0003916	0.900929
EVM0023918	EVM0002082	0.900929
EVM0005180	EVM0001739	0.900929
EVM0007391	EVM0027935	0.900929
EVM0009174	EVM0002082	0.900929
EVM0028138	Colocasia_escule	0.900929
EVM0016224	EVM0016563	0.900929
EVM0007214	EVM0025980	0.900929
EVM0027189	EVM0024069	0.900929
EVM0000767	EVM0002082	0.900929
EVM0023918	EVM0010332	0.900929
EVM0003656	EVM0008519	0.900929
EVM0007391	EVM0000767	0.900929
EVM0016224	EVM0010086	0.900929
EVM0013713	EVM0010982	0.900929
EVM0016224	Colocasia_escule	0.900929
EVM0007214	EVM0020308	0.900929
EVM0014597	EVM0007214	0.900929
EVM0000765	Colocasia_escule	0.900929
Colocasia_esculenta_newGene_956	EVM0016728	0.900929
EVM0016563	EVM0023082	0.900929
EVM0006304	EVM0024682	0.900929
EVM0016563	EVM0009322	0.900929
EVM0022133	EVM0002433	0.900929
EVM0023082	EVM0020564	0.900929
EVM0023082	EVM0008570	0.900929
EVM0023082	EVM0010982	0.900929
EVM0011820	EVM0022327	0.900929
EVM0013047	EVM0014706	0.900929

EVM0013047	EVM0005432	0.900929
EVM0010536	EVM0013500	0.900929
EVM0014895	EVM0008948	0.900929
EVM0012409	EVM0020593	0.900929
EVM0016596	EVM0016010	0.900929
EVM0021390	EVM0018701	0.900929
EVM0025117	EVM0000765	0.900929
EVM0020564	EVM0012650	0.900929
EVM0020564	EVM0018701	0.900929
EVM0012316	EVM0007856	0.900929
EVM0027935	EVM0018490	0.900929
EVM0027935	EVM0002082	0.900929
EVM0027407	EVM0003916	0.900929
EVM0012316	EVM0016563	0.900929
EVM0012316	EVM0015270	0.900929
EVM0012316	EVM0007649	0.900929
EVM0000541	EVM0008570	0.900929
EVM0023938	EVM0020012	0.900929
EVM0021407	EVM0028138	0.900929
EVM0000541	EVM0007856	0.900929
EVM0021407	EVM0019309	0.900929
Colocasia_esculenta_newGene_462	EVM0024682	0.900929
Colocasia_esculenta_newGene_863	EVM0023082	0.900929
EVM0015270	EVM0018643	0.900929
EVM0015270	EVM0009554	0.900929
Colocasia_esculenta_newGene_239	EVM0012655	0.900929
Colocasia_esculenta_newGene_863	EVM0006551	0.900929
EVM0004594	EVM0002524	0.900929
EVM0004594	Colocasia_escule	0.900929
EVM0023059	EVM0020593	0.900929
EVM0023059	EVM0001853	0.900929
EVM0020012	EVM0008519	0.900929
EVM0026177	EVM0005180	0.900929
EVM0020012	EVM0002082	0.900929
EVM0020012	EVM0023403	0.900929
EVM0005432	EVM0024474	0.900929
EVM0012695	EVM0000765	0.900929
EVM0022436	EVM0006551	0.900929
EVM0012695	EVM0004228	0.900929
EVM0012695	EVM0016563	0.900929
EVM0024474	EVM0000541	0.900929
EVM0019373	EVM0027715	0.900929
EVM0024474	EVM0024069	0.900929
EVM0016010	EVM0016563	0.900929
EVM0027553	EVM0021848	0.900929
EVM0001853	EVM0023082	0.900929
EVM0002590	EVM0002433	0.900929
EVM0002590	EVM0018411	0.900929
EVM0001853	EVM0020593	0.900929
EVM0001853	EVM0018643	0.900929
EVM0002590	EVM0001853	0.900929
EVM0027715	EVM0016606	0.900929
EVM0027715	EVM0018411	0.900929
EVM0008474	Colocasia_escule	0.900929
EVM0010086	EVM0000767	0.900929
EVM0000811	EVM0002433	0.900929
EVM0010086	EVM0012316	0.900929
Colocasia_esculenta_newGene_865	EVM0008474	0.900929

EVM0002433	EVM0020628	0.900929
Colocasia_esculenta_newGene_901	EVM0023576	0.900929
Colocasia_esculenta_newGene_901	Colocasia_escule	0.900929
EVM0027449	EVM0011632	0.900929
EVM0015080	EVM0015270	0.900929
EVM0015080	EVM0025980	0.900929
EVM0008570	EVM0018643	0.900929
EVM0016728	EVM0006551	0.900929
EVM0016728	EVM0007856	0.900929
EVM0020216	EVM0022715	0.900929
EVM0024682	EVM0023970	0.900929
EVM0020216	EVM0008210	0.900929
EVM0024682	EVM0006551	0.900929
EVM0024682	EVM0012655	0.900929
EVM0005581	EVM0028456	0.900929
EVM0020593	EVM0015709	0.900929
EVM0020593	EVM0008519	0.900929
EVM0014369	EVM0012650	0.900929
EVM0026948	EVM0008570	0.900929
EVM0018643	EVM0013669	0.900929
EVM0012607	EVM0005844	0.900929
Colocasia_esculenta_newGene_570	EVM0020748	0.900929
Colocasia_esculenta_newGene_570	EVM0002082	0.900929
EVM0012607	Colocasia_escule	0.900929
EVM0012607	EVM0011820	0.900929
EVM0012607	EVM0010086	0.900929
EVM0012607	Colocasia_escule	0.900929
EVM0021407	EVM0004228	0.900929
EVM0021407	EVM0005018	0.900929
EVM0005018	EVM0010982	0.900929
EVM0010905	EVM0018411	0.900929
EVM0021407	EVM0020012	0.900929
EVM0021407	EVM0005432	0.900929
EVM0021407	EVM0019373	0.900929
EVM0026358	EVM0003916	0.900929
EVM0005345	EVM0018490	0.900929
EVM0005345	EVM0026358	0.900929
EVM0005345	EVM0002590	0.900929
EVM0010332	EVM0027189	0.900929
EVM0010332	EVM0014706	0.900929
EVM0018411	EVM0001739	0.900929
EVM0018411	EVM0028206	0.900929
EVM0010332	EVM0002590	0.900929
EVM0010332	EVM0006304	0.900929
EVM0008602	EVM0007856	0.900929
EVM0003916	EVM0004228	0.900929
EVM0003916	EVM0006551	0.900929
Colocasia_esculenta_newGene_647	EVM0023059	0.900929
EVM0022715	EVM0006551	0.900929
EVM0018760	EVM0013713	0.900929
EVM0008210	EVM0028206	0.900929
EVM0027734	EVM0004228	0.900929
EVM0008210	EVM0010905	0.900929
EVM0008210	EVM0022133	0.900929
EVM0019229	EVM0020748	0.900929
EVM0019229	EVM0002433	0.900929
EVM0019229	EVM0027715	0.900929
EVM0008474	EVM0006551	0.900929

EVM0008474	EVM0015709	0.900929

Note: Degree>0.9
