# Supplementary material for: Analysis of Gene Regulatory Networks of Taro (Colocasia esculenta (L.) Schott.) Soluble Starch Synthase Based on DeGN and KASP Marker Development
Source: Int J Genomics. 2025 Mar 1;2025:9953367. doi: 10.1155/ijog/9953367 (PMC11991784; doi:10.1155/ijog/9953367)
Supplement: Supporting Information — Additional supporting information can be found online in the Supporting Information section. Table S1: List of 89 taro resources, original source, variety type, morphotype, phenotypic traits, and starch component contents. Table S2: All transcript expression value (FPKM) in taro corm developing stage T1 to T6. Table S3: Gene interactions between DEGs in taro corm developing stage. Table S4: Expression profile of CeSSI, CeSS II, CeMy108, and SerThr kinase. Table S5: The genotypes of 89 taro resources. Table S6: 159 node genes of CeSS regulatory network. [file 9953367.f1.zip › Supplementary Table S4. Expression profile of CeSS_, CeSS_, CeMy108 and SerThr kinase..pdf]

Supplementary Table S4. Expression profile of *CeSS I*, *CeSS II*, *CeMy108* and *Ser/Thr kinase*.

Gene Symbo	T1-1	T1-2	T1-3	T2-1	T2-2	T2-3	T3-1	T3-2	T3-3	T4-1	T4-2	T4-3	T5-1	T5-2	T5-3	T6-1	T6-2	T6-3
EVM0005345	34.05	38.14	41.9	55.63	61.3	67.79	46.09	17.88	39.96	12.72	10.47	9.998	10.59	17.49	8.104	17.54	22.7	18.32
EVM0022436	3.752	3.388	4.471	5.036	7.336	5.89	2.148	1.372	1.774	0.316	0.173	0.219	0.086	0	0.252	0.407	1.126	0.603
EVM0003916	4.706	7.222	5.13	2.079	2.882	2.198	3.986	8.141	3.899	19.92	32.85	31.09	34.52	23.7	27.68	21.01	11.7	17.68
EVM0025803	7.266	7.199	9.449	13.99	15.59	19.19	11.74	12.27	10.48	2.284	2.54	1.787	4.587	6.717	3.516	10.89	8.843	9.261
