# Supplementary material for: Analysis of Gene Regulatory Networks of Taro (Colocasia esculenta (L.) Schott.) Soluble Starch Synthase Based on DeGN and KASP Marker Development
Source: Int J Genomics. 2025 Mar 1;2025:9953367. doi: 10.1155/ijog/9953367 (PMC11991784; doi:10.1155/ijog/9953367)
Supplement: Supporting Information — Additional supporting information can be found online in the Supporting Information section. Table S1: List of 89 taro resources, original source, variety type, morphotype, phenotypic traits, and starch component contents. Table S2: All transcript expression value (FPKM) in taro corm developing stage T1 to T6. Table S3: Gene interactions between DEGs in taro corm developing stage. Table S4: Expression profile of CeSSI, CeSS II, CeMy108, and SerThr kinase. Table S5: The genotypes of 89 taro resources. Table S6: 159 node genes of CeSS regulatory network. [file 9953367.f1.zip › Supplementary Table S6. 159 node genes of CeSS regulatory network..pdf]

Supplementary Table S6. 159 node genes of CeSS regulatory network.

GeneID	COG_class	COG_class_annotation	GO_annotation	KEGG_annotation	KEGG_pathway_annotation	KOG_class	KOG_class_annotation	Pfam_annotation	Swiss-Prot_annotation	eggNOG_class	eggNOG_class_annotation	NR_annotation
EVM0000541	[P]	Inorganic ion transpo	Molecular Function: D	K01725|1.3e-68|jcu:10564	Nitrogen metabol	--	--	Cyanate lyase C-ter	Cyanate hydrat	--	--	Cyanase [Handroanthus impetiginosus]
EVM0000765	[QV]	Secondary metabolit	Molecular Function: n	K20617|2.7e-142|egu:105	--	[Q]	Secondary metabo	Cytochrome P450	Cytochrome P4	--	--	cytochrome P450 71A9-like protein [Cinnamomum micranthum f. kanehirae]
EVM0000767	--	--	Biological Process: int	--	--	[U]	Intracellular traffick	--	TOM1-like pro	--	--	target of Myb protein 1 [Cinnamomum micranthum f. kanehirae]
EVM0000811	--	--	--	--	--	--	--	--	--	--	--	PREDICTED: uncharacterized protein LOC104597064 [Nelumbo nucifera]
EVM0001096	[H]	Coenzyme transport	Molecular Function: C	K16040|3.1e-87|egu:1050	Stilbenoid, diarylh	[R]	General function pr	O-methyltransferase	8-hydroxyquer	--	--	trans-resveratrol di-O-methyltransferase [Elaeis guineensis]
EVM0001224	--	--	Cellular Component: i	--	--	--	--	--	--	--	--	uncharacterized protein LOC105032418 [Elaeis guineensis]
EVM0001281	--	--	Molecular Function: n	K17710|5.1e-11|gmx:1008	--	[R]	General function pr	PPR repeat family	Pentatricopept	--	--	putative Pentatricopeptide repeat-containing protein [Zostera marina]
EVM0001733	--	--	Cellular Component: e	K20628|4.7e-57|lsv:11187	--	--	--	Lytic transglycolase	Expansin-A13	--	--	expansin-A13 [Amorphophallus albus]
EVM0001739	--	--	--	K03257|1.0e-24|nta:10776	RNA transport (ko	--	--	--	Protein FLX-lik	--	--	hypothetical protein VITISV_027853 [Vitis vinifera]
EVM0001853	--	--	Biological Process: pro	K04797|1.3e-51|nnu:1045	--	[O]	Posttranslational m	Prefoldin subunit	Probable prefo	--	--	PREDICTED: probable prefoldin subunit 5 [Nelumbo nucifera]
EVM0001947	--	--	Biological Process: res	--	--	--	--	--	--	--	--	F-box protein At1g61340 [Elaeis guineensis]
EVM0002082	--	--	Molecular Function: p	K03122|7.9e-07|mdm:114	Basal transcription	--	--	PB1 domain	--	--	--	hypothetical protein C4D60_Mb11t11330 [Musa balbisiana]
EVM0002433	--	--	--	--	--	--	--	--	--	--	--	PREDICTED: uncharacterized protein LOC103978898 [Musa acuminata subsp. malaccensis]
EVM0002524	--	--	Cellular Component: i	K15100|3.2e-156|tcc:1860	--	[C]	Energy production	Mitochondrial carrie	Mitochondrial	--	--	Mitochondrial substrate/solute carrier [Parasponia andersonii]
EVM0002590	[Q]	Secondary metabolit	Molecular Function: o	K05278|8.0e-44|han:1108	Flavonoid biosynt	[QR]	Secondary metabo	non-haem dioxyger	Flavonol syntha	--	--	probable 2-oxoglutarate-dependent dioxygenase At5g05600 [Phoenix dactylifera]
EVM0003654	[QV]	Secondary metabolit	Molecular Function: n	K12639|1.3e-218|pda:103	Brassinosteroid bi	[QI]	Secondary metabo	Cytochrome P450	Cytochrome P4	--	--	cytochrome P450 724B1 [Phoenix dactylifera]
EVM0003916	--	--	Molecular Function: D	K09422|2.7e-92|pda:1037	--	[K]	Transcription	Myb-like DNA-binc	Transcription fa	--	--	transcription factor MYB62-like [Phoenix dactylifera]
EVM0004228	--	--	--	--	--	[BK]	Chromatin structure	Divergent CRAL/TRI	--	--	--	ganglioside-induced differentiation-associated protein 2 [Phoenix dactylifera]
EVM0004594	--	--	Cellular Component: i	--	--	[U]	Intracellular traffick	TLC domain	ASC1-like prot	--	--	LAG1 longevity assurance homolog 3 [Prunus persica]
EVM0005018	[H]	Coenzyme transport	Molecular Function: C	K16040|1.4e-51|pper:109	Stilbenoid, diarylh	[R]	General function pr	O-methyltransferase	Acetylserotonin	--	--	trans-resveratrol di-O-methyltransferase [Dorcoceras hygrometricum]
EVM0005180	--	--	Cellular Component: i	K21989|1.1e-260|egu:105	--	[R]	General function pr	Calcium-dependent	Protein OSCA1	--	--	calcium permeable stress-gated cation channel 1 isoform X1 [Elaeis guineensis]
EVM0005345	[G]	Carbohydrate transp	Molecular Function: g	K00703|5.4e-278|egu:105	Starch and sucros	--	--	Starch synthase cata	Starch synthase	--	--	soluble starch synthase II [Colocasia esculenta]
EVM0005432	[C]	Energy production ar	Molecular Function: tr	K09699|2.2e-179|egu:105	Valine, leucine an	[C]	Energy production	2-oxoacid dehydrog	Lipoamide acy	--	--	lipoamide acyltransferase component of branched-chain alpha-keto acid dehydrogenase complex, mitochondrial isoform X1 [Elaeis guineensis]
EVM0005581	[O]	Posttranslational mo	Molecular Function: A	K13525|9.0e-112|ccav:112	Protein processing	[O]	Posttranslational m	ATPase family assoc	Cell division co	--	--	cell division control protein 48 homolog B isoform X1 [Elaeis guineensis]
EVM0005844	--	--	Molecular Function: G	K15281|1.4e-81|spen:107	--	[GOU]	Carbohydrate trans	Triose-phosphate T	GDP-fucose tra	--	--	GDP-mannose transporter GONST4 [Asparagus officinalis]
EVM0006304	--	--	Molecular Function: c	K08901|4.0e-85|dzi:11128	Photosynthesis (ko	--	--	Oxygen evolving en	Oxygen-evolvi	--	--	oxygen-evolving enhancer protein 3, chloroplastic-like [Durio zibethinus]
EVM0006551	[G]	Carbohydrate transp	Cellular Component: i	K08193|2.9e-142|ccav:112	--	[G]	Carbohydrate trans	Major Facilitator Sup	Probable anion	--	--	probable anion transporter 3, chloroplastic isoform X2 [Ananas comosus]
EVM0006897	--	--	Cellular Component: i	--	--	--	--	--	Protein EARLY	--	--	PREDICTED: polyadenylate-binding protein-interacting protein 2-like [Nelumbo nucifera]
EVM0007090	[G]	Carbohydrate transp	Molecular Function: A	K00975|4.4e-223|atr:1842	Starch and sucros	[M]	Cell wall/membrane	Nucleotidyl transfera	Glucose-1-pho	--	--	putative ADP-glucose pyrophosphorylase, partial [Amorphophallus konjac]
EVM0007214	[G]	Carbohydrate transp	Molecular Function: p	K01213|1.1e-17|bdi:10083	Pentose and gluc	--	--	Glycosyl hydrolases	--	--	--	putative polygalacturonase [Ananas comosus]
EVM0007391	--	--	Molecular Function: tr	K14432|8.0e-98|egu:1050	Plant hormone sig	--	--	bZIP transcription fa	ABSCISIC ACID	--	--	ABSCISIC ACID-INSENSITIVE 5-like protein 2 [Cinnamomum micranthum f. kanehirae]
EVM0007483	--	--	Molecular Function: li	--	--	--	--	Domain of unknown	--	--	--	Protein of unknown function DUF3741 [Macleaya cordata]
EVM0007532	[J]	Translation, ribosoma	Molecular Function: n	--	--	--	--	Ribosomal silencing	Protein lojap, c	--	--	Protein lojap/ribosomal silencing factor RsfS [Cinnamomum micranthum f. kanehirae]
EVM0007649	--	--	--	--	--	--	--	--	--	--	--	uncharacterized protein LOC103709757 isoform X2 [Phoenix dactylifera]
EVM0007856	[P]	Inorganic ion transpo	Molecular Function: c	K14689|1.8e-145|pda:103	--	[P]	Inorganic ion trans	Cation efflux family	Metal toleranc	--	--	metal tolerance protein 1-like [Phoenix dactylifera]
EVM0008210	[T]	Signal transduction n	Molecular Function: p	K00898|9.1e-180|mus:103	--	[T]	Signal transduction	Mitochondrial branc	[Pyruvate dehy	--	--	PREDICTED: pyruvate dehydrogenase (acetyl-transferring) kinase, mitochondrial [Musa acuminata subsp. malaccensis]
EVM0008372	--	--	--	--	--	--	--	--	Putative diseas	S	Function unknow	PREDICTED: uncharacterized protein [Pyrus x bretschneideri]
EVM0008474	--	--	Cellular Component: i	--	--	--	--	Late embryogenesis	NDR1/HIN1-lik	--	--	NDR1/HIN1-like protein 1 [Elaeis guineensis]
EVM0008519	--	--	Molecular Function: D	--	--	[KB]	Transcription;; Chro	Methyl-CpG binding	Methyl-CpG-b	--	--	methyl-CpG-binding domain-containing protein 4-like [Panicum hallii]
EVM0008570	--	--	Cellular Component: i	K21989|4.3e-289|egu:105	--	[R]	General function pr	Calcium-dependent	CSC1-like prot	--	--	calcium permeable stress-gated cation channel 1 isoform X3 [Elaeis guineensis]
EVM0008602	--	--	Biological Process: res	--	--	--	--	--	Protein PROTC	--	--	hypothetical protein BVC80_9099g133 [Macleaya cordata]
EVM0008948	--	--	Cellular Component: c	K01304|1.5e-97|pda:1037	--	[O]	Posttranslational m	Pyroglutamyl peptic	--	--	--	uncharacterized protein LOC103700943 [Phoenix dactylifera]
EVM0009174	--	--	--	--	--	--	--	Fasciclin domain	Fasciclin-like a	--	--	fasciclin-like arabinogalactan protein 2 [Phoenix dactylifera]
EVM0009233	--	--	Cellular Component: i	--	--	--	--	Protein of unknown	Tobamovirus n	--	--	PREDICTED: tobamovirus multiplication protein 1 [Theobroma cacao]
EVM0009322	--	--	--	--	--	--	--	C2 domain	--	--	--	hypothetical protein CMV_017246 [Castanea mollissima]
EVM0009531	[Q]	Secondary metabolit	Molecular Function: la	K01759|8.0e-168|nnu:104	Pyruvate metabol	[G]	Carbohydrate trans	Glyoxalase/Bleomyc	Probable lacto	--	--	PREDICTED: probable lactoylglutathione lyase, chloroplastic [Nelumbo nucifera]
EVM0009554	--	--	--	--	--	--	--	Protein of unknown	--	--	--	hypothetical protein C4D60_Mb01t01010 [Musa balbisiana]
EVM0009588	[L]	Replication, recombi	Molecular Function: n	K11594|1.3e-100|jcu:1056	--	[A]	RNA processing an	Helicase conserved	DEAD-box ATI	--	--	DEAD-box ATP-dependent RNA helicase 37 [Jatropha curcas]

EVM0010086	--	--	Cellular Component:	K12125|2.3e-137|egu:105	Circadian rhythm	--	--	--	ELF3-like prote	--	--	protein HEADING DATE 3B [Elaeis guineensis]
EVM0010332	--	--	Biological Process: mF	K13153|1.7e-65|nnu:1045	--	--	--	Ubiquitin-like doma	U11/U12 small	--	--	U11/U12 small nuclear ribonucleoprotein 25 kDa protein isoform X3 [Elaeis guineensis]
EVM0010530	--	--	Molecular Function: R	--	--	--	--	OST-HTH/LOTUS d	Zinc finger CC	--	--	PREDICTED: zinc finger CCCH domain-containing protein 18 [Nelumbo nucifera]
EVM0010536	[R]	General function pre	Molecular Function: n	K08999|5.0e-33|ppp:1122	--	--	--	Domain of unknowr	Bifunctional nu	--	--	bifunctional nuclease 2 [Cinnamomum micranthum f. kanehirae]
EVM0010905	--	--	Molecular Function: n	K10406|1.7e-76|tcc:18611	--	[N]	Cell motility	Kinesin motor doma	Kinesin-like pr	--	--	kinesin-like protein KIN-14E isoform X1 [Cinnamomum micranthum f. kanehirae]
EVM0010982	[Q]	Secondary metabolit	Molecular Function: o	K05278|2.2e-36|han:1108	Flavonoid biosynt	[QR]	Secondary metabo	non-haem dioxyger	Protein SRG1 (	--	--	protein SRG1 isoform X1 [Elaeis guineensis]
EVM0011330	--	--	Cellular Component: i	--	--	--	--	--	--	--	--	uncharacterized protein Pyn_17115 [Prunus yedoensis var. nudiflora]
EVM0011473	--	--	--	K01179|4.0e-56|ccav:1125	Starch and sucros	--	--	Leucine rich repeat	--	--	--	putative adenylate cyclase regulatory protein isoform X1 [Phoenix dactylifera]
EVM0011632	--	--	Molecular Function: D	K08869|6.0e-22|cre:CHLRE	--	--	--	Myb-like DNA-binc	Probable trans	--	--	transcription factor KUA1 [Elaeis guineensis]
EVM0011820	[QV]	Secondary metabolit	Molecular Function: n	K20617|6.2e-144|egu:105	--	[Q]	Secondary metabo	Cytochrome P450	Cytochrome P4	--	--	cytochrome P450 71A9-like protein [Cinnamomum micranthum f. kanehirae]
EVM0012316	--	--	Molecular Function: h	K01206|1.0e-136|thj:1048	Other glycan deg	--	--	GDSL-like Lipase/Ac	GDSL esterase/	--	--	GDSL esterase/lipase At3g26430-like isoform X1 [Phoenix dactylifera]
EVM0012360	--	--	--	--	--	--	--	Protein of unknown	--	--	--	PREDICTED: uncharacterized protein LOC108211480 [Daucus carota subsp. sativus]
EVM0012409	[G]	Carbohydrate transp	Molecular Function: n	K01835|8.8e-284|jcu:1056	Glycolysis / Gluco	[G]	Carbohydrate trans	Phosphoglucomuta	Phosphogluco	--	--	PGM_PMM_IV domain-containing protein/PGM_PMM_I domain-containing protein/PGM_PMM_II domain-containing protein/PGM_PMM_III domain-containing protein [Cephalotus follicularis]
EVM0012607	--	--	Molecular Function: tr	--	--	[K]	Transcription	SSXT protein (N-ter	GRF1-interacti	--	--	GRF1-interacting factor 2-like [Phoenix dactylifera]
EVM0012650	--	--	Molecular Function: d	K15032|9.5e-111|egu:105	--	[KR]	Transcription;; Gen	mTERF	Transcription t	--	--	transcription termination factor MTERF5, chloroplastic [Ananas comosus]
EVM0012655	--	--	Molecular Function: z	K22378|9.4e-16|bna:1064	--	[O]	Posttranslational m	Ring finger domain	Probable E3 ub	--	--	probable E3 ubiquitin-protein ligase RHB1A [Phoenix dactylifera]
EVM0012695	--	--	--	K01191|1.9e-37|pxb:1039	Other glycan deg	--	--	Protein of unknown	--	--	--	hypothetical protein C1H46_044783 [Malus baccata]
EVM0013047	--	--	Cellular Component: I	--	--	--	--	--	Centromere pr	--	--	centromere protein C [Elaeis guineensis]
EVM0013500	--	--	--	--	--	--	--	--	--	--	--	uncharacterized protein LOC109012192 isoform X1 [Juglans regia]
EVM0013669	--	--	--	--	--	--	--	--	--	--	--	hypothetical protein GW17_00016539 [Ensete ventricosum]
EVM0013713	--	--	Molecular Function: tr	K14972|1.6e-63|cic:CICLE_	--	--	--	KIX domain	Mediator of RN	--	--	CTV.22 [Citrus trifoliata]
EVM0013823	[S]	Function unknown	Cellular Component:	K13449|9.0e-56|egu:1050	MAPK signaling p	[S]	Function unknown	Cysteine-rich secret	Pathogenesis-	--	--	pathogenesis-related protein PRB1-3-like protein [Cinnamomum micranthum f. kanehirae]
EVM0014369	--	--	Cellular Component: i	--	--	--	--	--	Signaling pepti	--	--	signaling peptide TAXIMIN 1 [Elaeis guineensis]
EVM0014578	--	--	Cellular Component: i	--	--	--	--	Plant phosphoribos	Protein QUIRK	--	--	hypothetical protein B296_00037094 [Ensete ventricosum]
EVM0014597	--	--	Cellular Component:	--	--	--	--	Domain of unknowr	CASP-like prot	--	--	CASP-like protein 1D1 [Phoenix dactylifera]
EVM0014706	[QV]	Secondary metabolit	Molecular Function: n	K09588|5.9e-162|pda:103	Brassinosteroid bi	[QI]	Secondary metabo	Cytochrome P450	Cytochrome P4	--	--	cytochrome P450 90A1-like protein [Cinnamomum micranthum f. kanehirae]
EVM0014742	--	--	Molecular Function: D	K10882|2.3e-151|pda:103	Homologous recc	--	--	ERCC4 domain	Crossover junc	--	--	crossover junction endonuclease EME1B-like isoform X1 [Phoenix dactylifera]
EVM0014895	[G]	Carbohydrate transp	Molecular Function: A	K00975|1.1e-250|nnu:104	Starch and sucros	[M]	Cell wall/membran	Nucleotidyl transfer	Glucose-1-ph	--	--	ADP-glucose pyrophosphorylase 1 [Colocasia esculenta]
EVM0015080	[QV]	Secondary metabolit	Molecular Function: ir	K20495|7.8e-127|ppp:112	Fatty acid degrad	[QI]	Secondary metabo	Cytochrome P450	Cytochrome P4	--	--	cytochrome P450 704C1-like [Phoenix dactylifera]
EVM0015270	--	--	--	--	--	--	--	--	--	--	--	PREDICTED: uncharacterized protein LOC104597064 [Nelumbo nucifera]
EVM0015541	[J]	Translation, ribosom	Molecular Function: n	K19589|2.8e-104|egu:105	--	[J]	Translation, ribosor	Methyltransferase sı	--	--	--	methyltransferase N6AMT1 isoform X1 [Elaeis guineensis]
EVM0015709	--	--	Biological Process: int	K13141|2.1e-49|csat:1047	--	[U]	Intracellular traffick	VHS domain	TOM1-like pro	--	--	LOW QUALITY PROTEIN: TOM1-like protein 5 [Elaeis guineensis]
EVM0016008	--	--	Molecular Function: A	--	--	--	--	Plants and Prokaryo	AT-hook motif	--	--	AT-hook motif nuclear-localized protein 1 [Elaeis guineensis]
EVM0016010	[O]	Posttranslational mo	Molecular Function: A	K01338|0.0e+00|pda:1037	--	[O]	Posttranslational m	Lon protease (S16)	Lon protease h	--	--	LOW QUALITY PROTEIN: lon protease homolog 2, peroxisomal-like [Phoenix dactylifera]
EVM0016156	[G]	Carbohydrate transp	Cellular Component: i	K14445|1.2e-199|dct:1100	--	[P]	Inorganic ion trans	Sodium:sulfate sym	Tonoplast dica	--	--	tonoplast dicarboxylate transporter isoform X1 [Dendrobium catenatum]
EVM0016194	[G]	Carbohydrate transp	Molecular Function: p	K01184|2.1e-32|brp:1038	Pentose and gluc	--	--	Glycosyl hydrolases	Probable polyg	--	--	probable polygalacturonase [Phoenix dactylifera]
EVM0016224	[IQR]	Lipid transport and m	Cellular Component: i	K11170|5.6e-83|lang:1093	--	[Q]	Secondary metabo	short chain dehydro	Short-chain de	--	--	dehydrogenase/reductase SDR family member on chromosome X [Elaeis guineensis]
EVM0016563	--	--	Cellular Component: i	K21989|1.6e-100|egu:105	--	[R]	General function p	Calcium-dependent	Protein OSCA1	--	--	calcium permeable stress-gated cation channel 1 isoform X1 [Elaeis guineensis]
EVM0016596	--	--	Cellular Component: i	--	--	--	--	Transmembrane pr	Protein FATTY	--	--	PREDICTED: protein FATTY ACID EXPORT 3, chloroplastic [Nelumbo nucifera]
EVM0016606	--	--	--	K13963|1.6e-27|vra:11124	--	--	--	FAR1 DNA-binding	Protein FAR1-I	--	--	uncharacterized protein LOC105043030 [Elaeis guineensis]
EVM0016728	--	--	--	--	--	--	--	--	Uncharacterize	--	--	
EVM0017284	--	--	Molecular Function: D	--	--	--	--	Zinc knuckle	--	--	--	uncharacterized protein LOC110106648 [Dendrobium catenatum]
EVM0017550	[C]	Energy production a	Molecular Function: o	K12355|2.3e-220|egu:105	Phenylpropanoid	[C]	Energy production	Aldehyde dehydrog	Aldehyde dehy	--	--	aldehyde dehydrogenase family 2 member C4 [Elaeis guineensis]
EVM0018411	[K]	Transcription	Molecular Function: tr	K03093|7.4e-149|pda:103	--	--	--	Sigma-70, region 4	RNA polymera	--	--	RNA polymerase sigma factor sigC isoform X1 [Phoenix dactylifera]
EVM0018490	[J]	Translation, ribosom	Biological Process: tR	K10760|6.2e-89|fve:10129	Zeatin biosynthes	[J]	Translation, ribosor	IPP transferase	Adenylate isop	--	--	tRNA isopentenyltransferase [Macleaya cordata]
EVM0018643	[QV]	Secondary metabolit	Molecular Function: ir	K20617|2.7e-113|egu:105	--	[Q]	Secondary metabo	Cytochrome P450	Cytochrome P4	--	--	cytochrome P450 71A1-like [Phoenix dactylifera]
EVM0018701	[V]	Defense mechanisms	Molecular Function: a	K03327|6.9e-186|pda:103	--	[R]	General function p	MatE	Protein DETOX	--	--	protein DETOXIFICATION 16-like [Phoenix dactylifera]
EVM0018760	[P]	Inorganic ion transp	Molecular Function: c	K17686|0.0e+00|pda:1037	MAPK signaling p	[P]	Inorganic ion trans	E1-E2 ATPase	Copper-transp	--	--	copper-transporting ATPase HMA4-like [Phoenix dactylifera]
EVM0019229	--	--	Cellular Component: i	--	--	--	--	Aluminium induced	Stem-specific	--	--	aluminum-induced protein [Cinnamomum micranthum f. kanehirae]
EVM0019309	--	--	--	--	--	--	--	--	--	--	--	hypothetical protein COO60DRAFT_1091220 [Scenedesmus sp. NREL 46B-D3
EVM0019373	--	--	Molecular Function: A	--	--	--	--	Plants and Prokaryo	AT-hook motif	--	--	AT-hook motif nuclear-localized protein 10 [Elaeis guineensis]
EVM0019956	--	--	Molecular Function: tr	K20557|1.8e-42|nnu:1045	MAPK signaling p	--	--	bZIP transcription fa	bZIP transcript	--	--	Basic-leucine zipper domain [Macleaya cordata]
EVM0020012	[QV]	Secondary metabolit	Molecular Function: n	K07425|2.2e-155|ccaj:109	Fatty acid degrad	[QI]	Secondary metabo	Cytochrome P450	Cytochrome P4	--	--	cytochrome P450 CYP72A219-like [Phoenix dactylifera]

EVM0020216	[P]	Inorganic ion transp	Molecular Function: p	K06195|4.1e-84|ccaj:1098	--	--	--	ApaG domain	--	--	--	uncharacterized protein LOC103724177 isoform X1 [Phoenix dactylifera]
EVM0020308	[MR]	Cell wall/membrane/	Molecular Function: A	--	--	--	--	D-ala D-ala ligase	--	--	--	D-alanine--D-alanine ligase [Macleaya cordata]
EVM0020381	--	--	Molecular Function: p	K21843|1.9e-211|pda:103	--	[T]	Signal transduction	Tetratricopeptide re	Protein NPGR2	--	--	protein NPGR2 [Phoenix dactylifera]
EVM0020564	--	--	Molecular Function: p	K22484|5.1e-30|pvu:PHA	--	--	--	Helix-loop-helix D	Transcription f	--	--	transcription factor bHLH30-like [Phoenix dactylifera]
EVM0020593	--	--	Molecular Function: n	K10990|1.6e-31|mus:1040	--	--	--	RecQ mediated gen	RecQ-mediate	--	--	recQ-mediated genome instability protein 1 [Cinnamomum micranthum f. kanehirae]
EVM0020628	--	--	--	--	--	--	--	--	--	--	--	PREDICTED: uncharacterized protein LOC104597064 [Nelumbo nucifera]
EVM0020748	--	--	Molecular Function: D	K14491|1.6e-19|mus:1039	Plant hormone si	--	--	DNA-binding doma	BOA OS=Arab	--	--	DNA binding[Phoenix dactylifera]
EVM0021380	--	--	Biological Process: pr	K18789|1.3e-121|pda:103	--	[GMW]	Carbohydrate trans	Exostosin family	Probable glyco	--	--	probable glycosyltransferase At5g03795 [Asparagus officinalis]
EVM0021390	--	--	Molecular Function: p	--	--	--	--	Leucine rich repeat	Uncharacterize	--	--	PREDICTED: leucine-rich repeat extensin-like protein 4 [Musa acuminata subsp. malaccensis]
EVM0021407	--	--	--	--	--	--	--	--	--	--	--	PREDICTED: uncharacterized protein LOC104597064 [Nelumbo nucifera]
EVM0021848	--	--	--	--	--	--	--	N-terminal C2 in EE	--	--	--	myosin-J heavy chain isoform X1 [Phoenix dactylifera]
EVM0022133	--	--	Biological Process: mi	--	--	[T]	Signal transduction	Leucine Rich repeat	--	--	--	uncharacterized protein LOC103701548 [Phoenix dactylifera]
EVM0022327	--	--	--	--	--	--	--	--	Protein NARR	--	--	hypothetical protein CKAN_01064200 [Cinnamomum micranthum f. kanehirae]
EVM0023059	[M]	Cell wall/membrane/	Molecular Function: c	K13082|1.0e-143|egu:105	Flavonoid biosynt	[V]	Defense mechanis	NAD dependent ep	Dihydroflavon	--	--	dihydroflavonol 4-reductase [Anthurium andraeanum]
EVM0022715	[C]	Energy production a	Molecular Function: N	K03809|3.6e-89|vvi:10025	Ubiquinone and c	[R]	General function p	NADPH-dependent	Probable NAD	--	--	PREDICTED: probable NAD(P)H dehydrogenase (quinone) FQR1-like 3 [Vitis vinifera]
EVM0023059	[M]	Cell wall/membrane/	Molecular Function: c	K13082|1.0e-143|egu:105	Flavonoid biosynt	[V]	Defense mechanis	NAD dependent ep	Dihydroflavon	--	--	dihydroflavonol 4-reductase [Anthurium andraeanum]
EVM0023082	--	--	Molecular Function: D	K00521|1.2e-36|cmos:111	--	--	--	No apical meristem	NAC domain-	--	--	NAC domain-containing protein 21/22 [Elaeis guineensis]
EVM0023403	[B]	Chromatin structure	Molecular Function: p	K15223|7.1e-70|pda:1037	--	[K]	Transcription	SWIB/MDM2 doma	--	--	--	upstream activation factor subunit spp27 isoform X1 [Cinnamomum micranthum f. kanehirae]
EVM0023576	--	--	Molecular Function: d	K15032|4.5e-129|egu:105	--	[KR]	Transcription;; Gen	mTERF	Transcription t	--	--	transcription termination factor MTERF2, chloroplastic isoform X1 [Elaeis guineensis]
EVM0023918	--	--	Molecular Function: r	K09060|1.2e-27|pop:1809	--	--	--	bZIP transcription f	G-box-binding	--	--	G-box-binding factor 1-like [Populus alba]
EVM0023938	[O]	Posttranslational mo	Molecular Function: t	K00799|1.7e-88|vvi:10026	Glutathione meta	[O]	Posttranslational m	Glutathione S-trans	Probable gluta	--	--	glutathione S-transferase U19 [Cannabis sativa]
EVM0023970	--	--	Molecular Function: A	K05665|3.1e-79|aof:10982	ABC transporters	[Q]	Secondary metabo	--	ABC transporte	--	--	ATP-binding cassette sub-family C member 8 [Crocus sativus]
EVM0024069	[G]	Carbohydrate transp	Cellular Component: i	K03319|7.3e-120|sly:1012	--	--	--	Sodium:sulfate sym	Dicarboxylate t	--	--	dicarboxylate transporter 1, chloroplastic [Cinnamomum micranthum f. kanehirae]
EVM0024351	--	--	Biological Process: fu	K23280|1.3e-78|lsv:11190	--	--	--	GDP-fucose protein	O-fucosyltrans	--	--	GDP-fucose protein O-fucosyltransferase [Macleaya cordata]
EVM0024474	[HT]	Coenzyme transport	Molecular Function: p	K08869|0.0e+00|aof:1098	--	[R]	General function p	ABC1 family	Protein ACTIVI	--	--	uncharacterized protein LOC105055798 isoform X2 [Elaeis guineensis]
EVM0024682	[HC]	Coenzyme transport	Molecular Function: s	K00511|3.9e-156|egu:105	Steroid biosynthe	[I]	Lipid transport and	Squalene epoxidase	Squalene epox	--	--	squalene monooxygenase [Elaeis guineensis]
EVM0025117	--	--	Cellular Component: i	K03377|1.7e-251|nnu:104	--	[R]	General function p	10 TM Acyl Transfer	Protein REDUC	--	--	Cas1p 10 TM acyl transferase domain [Macleaya cordata]
EVM0025628	--	--	Molecular Function: n	--	--	--	--	--	Ricin B-like lec	--	--	ricin B-like protein lectin R40G3 [Cinnamomum micranthum f. kanehirae]
EVM0025803	[G]	Carbohydrate transp	Molecular Function: g	K00703|4.2e-257|pda:103	Starch and sucros	--	--	Starch synthase cat	Soluble starch	--	--	soluble starch synthase I [Colocasia esculenta]
EVM0025980	--	--	Biological Process: re	--	--	--	--	--	Phytochrome	--	--	PREDICTED: phytochrome A-associated F-box protein [Musa acuminata subsp. malaccensis]
EVM0026115	[R]	General function pre	Cellular Component:	K01456|7.8e-21|pavi:1107	Protein processin	[C]	Energy production	Aldo/keto reductase	Probable volta	--	--	probable voltage-gated potassium channel subunit beta [Jatropha curcas]
EVM0026177	--	--	Molecular Function: R	--	--	--	--	Leucine rich repeat	Leucine-rich re	--	--	hypothetical protein C4D60_Mb06t06140 [Musa balbisiana]
EVM0026358	[G]	Carbohydrate transp	Cellular Component: i	K24193|1.5e-249|mus:103	--	[R]	General function p	Sugar (and other) tr	Sugar carrier p	--	--	hypothetical protein C4D60_Mb05t13370 [Musa balbisiana]
EVM0026363	--	--	Cellular Component:	K01280|1.6e-94|spen:107	--	[M]	Cell wall/membran	Pectinacetylesterase	Pectin acetyles	--	--	pectin acetylesterase 8-like isoform X1 [Phoenix dactylifera]
EVM0026948	[HC]	Coenzyme transport	Molecular Function: s	K00511|7.0e-228|egu:105	Steroid biosynthe	[I]	Lipid transport and	Squalene epoxidase	Squalene mon	--	--	squalene monooxygenase-like [Ananas comosus]
EVM0027189	[G]	Carbohydrate transp	Molecular Function: c	K16055|0.0e+00|nnu:1046	Starch and sucros	[G]	Carbohydrate trans	Glycosyltransferase	Alpha,alpha-tr	--	--	PREDICTED: alpha,alpha-trehalose-phosphate synthase [UDP-forming] 6-like [Nelumbo nucifera]
EVM0027407	--	--	Molecular Function: t	K20240|8.2e-125|jre:1089	--	--	--	Transferase family	Spermidine co	--	--	hypothetical protein F2P56_032229, partial [Juglans regia]
EVM0027449	--	--	Molecular Function: p	K01179|1.4e-38|ccav:1125	Starch and sucros	--	--	Leucine Rich repeat	--	--	--	putative adenylate cyclase regulatory protein isoform X1 [Phoenix dactylifera]
EVM0027553	--	--	Molecular Function: t	K14972|1.1e-62|mus:1039	--	--	--	KIX domain	Mediator of R	--	--	PREDICTED: mediator of RNA polymerase II transcription subunit 15a-like isoform X1 [Musa acuminata subsp. malaccensis]
EVM0027715	[G]	Carbohydrate transp	Molecular Function: c	K16055|4.2e-28|gab:1084	Starch and sucros	[G]	Carbohydrate trans	Trehalose-phospha	Alpha,alpha-tr	--	--	Alpha,alpha-trehalose-phosphate synthase 5 [Hibiscus syriacus]
EVM0027734	--	--	Biological Process: po	--	--	--	--	--	CCG-binding	--	--	CCG-binding protein 1 [Elaeis guineensis]
EVM0027935	--	--	Cellular Component: i	--	--	[P]	Inorganic ion trans	SPX domain	SPX domain-c	--	--	SPX domain-containing protein 4 isoform X2 [Phoenix dactylifera]
EVM0028138	--	--	--	--	--	--	--	--	--	--	--	hypothetical protein ACMD2_09467 [Ananas comosus]
EVM0028206	--	--	Biological Process: re	--	--	--	--	--	--	--	--	cyclin-dependent protein kinase inhibitor SMR15-like [Cucurbita pepo subsp. pepo]
EVM0028456	--	--	Molecular Function: t	K21383|4.3e-70|sind:1051	Anthocyanin bios	--	--	Transferase family	Malonyl-coen	--	--	anthocyanin 5-aromatic acyltransferase [Elaeis guineensis]
Colocasia_esculen	--	--	--	--	--	--	--	--	--	--	--	hypothetical protein COO60DRAFT_1091220 [Scenedesmus sp. NREL 46B-D3]
Colocasia_esculen	--	--	Molecular Function: n	--	--	--	--	--	Endonuclease	L	Replication, reco	PREDICTED: uncharacterized protein LOC105976358 [Erythranthe guttata]
Colocasia_esculen	--	--	--	--	--	--	--	--	--	--	--	PREDICTED: uncharacterized protein LOC104597064 [Nelumbo nucifera]
Colocasia_esculen	--	--	--	--	--	--	--	--	--	--	--	PREDICTED: uncharacterized protein LOC103978898 [Musa acuminata subsp. malaccensis]
Colocasia_esculen	--	--	--	--	--	--	--	--	Imidazole glyc	S	Function unknown	
Colocasia_esculen	--	--	--	--	--	--	--	--	Uncharacterize	--	--	
Colocasia_esculen	--	--	Molecular Function: s	--	--	--	--	--	--	--	--	rhomboid protein Pintu_RBL10, partial [Pinellia ternata]

Colocasia_esculen	[H]	Coenzyme transport	Molecular Function: C	K16040|1.8e-70|nsy:10422	Stilbenoid, diarylh	[R]	General function pr	O-methyltransferas	8-hydroxyquer	S	Function unknow	PREDICTED: trans-resveratrol di-O-methyltransferase-like [Nicotiana sylvestris]
Colocasia_esculen	--	--	--	--	--	--	--	--	Os04g068375	--	--	Os04g0683750 [Oryza sativa Japonica Group]
Colocasia_esculen	--	--	--	--	--	--	--	--	Coproporphyri	--	--	--
Colocasia_esculen	--	--	--	--	--	--	--	--	Uncharacterize	--	--	hypothetical protein MtrunA17_Chr8g0378081 [Medicago truncatula]
Colocasia_esculen	--	--	--	--	--	--	--	--	--	--	--	PREDICTED: uncharacterized protein LOC104597064 [Nelumbo nucifera]
Colocasia_esculen	--	--	--	--	--	--	--	--	--	--	--	PREDICTED: uncharacterized protein LOC103978898 [Musa acuminata subsp. malaccensis]
